# Hepatocyte-derived Igκ promotes HCC progression by stabilizing electron transfer flavoprotein subunit α to facilitate fatty acid β-oxidation

**DOI:** 10.1186/s13046-024-03203-8

**Published:** 2024-10-09

**Authors:** Jingjing Guo, Huining Gu, Sha Yin, Jiongming Yang, Qianqian Wang, Weiyan Xu, Yifan Wang, Shenghua Zhang, Xiaofeng Liu, Xunde Xian, Xiaoyan Qiu, Jing Huang

**Affiliations:** 1 Department of Immunology, School of Basic Medical Sciences, NHC Key Laboratory of Medical Immunology, Peking University, Beijing, 100191 China; 2 PUHSC Primary Immunodeficiency Research Center, Peking University, Beijing, 100191 China; 3 Heatopancreatobiliary Surgery Department I, Key Laboratory of Carcinogenesis and Translational Research (Ministry of Education/Beijing), Peking University Cancer Hospital & Institute, Beijing, 100142 China; 4 Institute of Cardiovascular Sciences, State Key Laboratory of Vascular Homeostasis and Remodeling, School of Basic Medical Sciences, Peking University, Beijing, 100191 China

## Abstract

**Background:**

Lipid metabolism dysregulation is a key characteristic of hepatocellular carcinoma (HCC) onset and progression. Elevated expression of immunoglobulin (Ig), especially the Igκ free light chain with a unique Vκ4-1/Jκ3 rearrangement in cancer cells, is linked to increased malignancy and has been implicated in colon cancer tumorigenesis. However, the role of Igκ in HCC carcinogenesis remains unclear. The aim of this study was to elucidate the pivotal roles of hepatocyte-derived Igκ in HCC development.

**Methods:**

The rearrangement sequence and expression level of hepatocyte-derived Igκ in HCC cells were determined via RT-PCR, Sanger sequencing, immunohistochemistry, and western blot analysis. The function of Igκ in HCC tumorigenesis was assessed by silencing Igκ using siRNA or gRNA in various HCC cell lines. To assess the role of Igκ in HCC pathogenesis in vivo, a mouse model with hepatocyte-specific Igκ knockout and diethylnitrosamine (DEN) and carbon tetrachloride (CCL4)-induced HCC was utilized. The molecular mechanism by which Igκ affects HCC tumorigenesis was investigated through multiomics analyses, quantitative real-time PCR, immunoprecipitation, mass spectrometry, immunofluorescence, and metabolite detection.

**Results:**

We confirmed that Igκ, especially Vκ4-1/Jκ3-Igκ, is highly expressed in human HCC cells. Igκ depletion inhibited HCC cell proliferation and migration in vitro, and hepatocyte-specific Igκ deficiency ameliorated HCC progression in mice with DEN and CCL4-induced HCC in vivo. Mechanistically, Vκ4-1/Jκ3-Igκ interacts with electron transfer flavoprotein subunit α (ETFA), delaying its protein degradation. Loss of Igκ led to a decrease in the expression of mitochondrial respiratory chain complexes III and IV, resulting in aberrant fatty acid β-oxidation (FAO) and lipid accumulation, which in turn inhibited HCC cell proliferation and migration.

**Conclusion:**

Our findings indicate that the Igκ/ETFA axis deregulates fatty acid β-oxidation, contributing to HCC progression, which suggests that targeting fatty acid metabolism may be an effective HCC treatment strategy. The results of this study suggest that hepatocyte-derived Vκ4-1/Jκ3-Igκ may serve as a promising therapeutic target for HCC.

**Supplementary Information:**

The online version contains supplementary material available at 10.1186/s13046-024-03203-8.

## Background

It is widely accepted that immunoglobulins (Igs) are produced by B-lineage lymphocytes, which play a critical role in the immune response as antibody molecules. However, accumulating evidence indicates that Igs can be widely produced by non-B cells, and these Igs are called non-B Igs and have activities distinct from those of antibodies, extending the scope of Ig function beyond humoral immunity. Over the past decades, our group and others have confirmed that normal non-B cells, including epithelial cells, cardiomyocytes, myeloid cells, spermatogenic cells, and neurons [1–5], especially a variety of epithelial tumor cells, can express high levels of Igs that promote cancer progression, metastasis, and drug resistance [6–11].

Generally, B-cell-derived Igs exhibit remarkable diversity due to Ig gene rearrangement. However, compared with normal B-cell-derived Igs, tumor-derived Igs often display a lower degree of variability. Our previous studies unexpectedly revealed that the Igκ light chain with a unique Vκ4-1/Jκ3 rearrangement pattern is widely expressed in different lineages of cancer cells, such as breast cancer, colon cancer, and lung squamous cell carcinoma cells and several other cancer cell lines [12]. Moreover, the tumor cell-derived Vκ4-1/Jκ3 light chain is typically highly homogeneous, with a pathogenic free Igκ light chain in amyloid light chain amyloidosis (AL-AM) and light chain deposition disease (LCDD) [13]. Vκ4-1/Jκ3-Igκ has been proven to serve as a novel ECM protein and integrin β1 ligand; specifically, it binds to integrin β1 and activates downstream FAK signalling to promote cancer cell proliferation and migration [12]. High expression of Igκ is closely related to a poor prognosis for breast cancer [14]. Therefore, it has been suggested that tumor-derived Igκ is closely associated with the development and metastasis of tumors and plays a pathogenic role. Igκ expression was first identified in primary hepatocytes from B-cell-deficient µMT mice and a normal liver cell line, and hepatocyte-derived Igκ expression was found to be increased during concanavalin A (ConA)-induced liver injury. More importantly, hepatocyte-specific deletion of Igκ exacerbated liver injury upon ConA challenge, suggesting that hepatocyte-derived Igκ is essential for hepatocyte survival [15].

Hepatocellular carcinoma (HCC), the most common type of liver cancer, accounting for approximately 90% of cases, and its incidence rate is higher in developing countries [16]; it is the sixth most common cancer and the fourth leading cause of cancer-related death worldwide [17]. Despite the application of various regimens in HCC treatment, tumor relapse occurs at a high rate (50-70% within 5 years after surgery), which limits improvements in survival; therefore, the prognosis of HCC is very dismal [18]. In addition, drug resistance remains the principal cause of targeted therapy treatment failure [19]. Thus, a comprehensive understanding of HCC tumorigenesis and identification of novel effective treatment targets for HCC are urgently needed.

Metabolic reprogramming is considered a major hallmark of tumorigenesis [20, 21]. Cancer cells often reprogram the metabolic pathways that control glycolysis, the tricarboxylic acid cycle (TCA), oxidative phosphorylation (OXPHOS), lipid synthesis, FAO, glutaminolysis, and mitochondrial metabolism during carcinogenesis. Alterations in fatty acid metabolism are increasingly recognized for their essential role in inducing HCC carcinogenesis [22]. However, whether the deregulation of FAO contributes to HCC tumorigenesis remains unclear, and this lack of clarity may be due to the tumor heterogeneity. Moreover, the expression of many β-oxidation-related genes varies significantly among various patients [23]. All of the above evidence highlights that targeting FAO is a highly promising strategy for treating HCC.

In recent years, a series of studies have revealed that tumor cell-derived IgG is involved in malignant tumor progression and that Vκ4-1/Jκ3-Igκ, a novel ECM protein, participates in colon cancer progression, but the role of hepatocyte-derived Igκ in HCC tumorigenesis has not been elucidated. Here, we confirmed the expression of Vκ4-1/Jκ3-Igκ in human HCC cells and demonstrated that hepatocyte-derived Igκ is involved in HCC progression. Mechanistically, we found for the first time that hepatocyte-derived Vκ4-1/Jκ3-Igκ interacts with the electron transporter ETFA on the mitochondrial respiratory chain to promote lipid metabolism and cell proliferation and migration in HCC, thereby promoting HCC tumorigenesis. Our findings reveal the key roles of hepatocyte-derived Igκ in HCC development and suggest its potential as a target for HCC treatment.

## Methods

### HCC patients and specimens

Human HCC tissues and adjacent non-tumor tissues were provided by Peking University Cancer Hospital (PUCH). All patients involved in this study had signed informed consent forms. The relevant clinical and histopathological data provided to researchers were anonymized. This study was approved by the Ethics Committee of PUCH.

### Cell culture

Human HCC cell lines (Huh7 and MHCC-97 H) were obtained from the PUCH. HEK293T cell line was purchased from American Type Culture Collection (ATCC) and maintained by the Peking University Center of Human Disease Genomics. All of these cell lines were cultured in Dulbecco’s Modified Eagle Medium (DMEM; Invitrogen, USA) supplemented with 10% Fetal Bovine Serum (FBS; PAN Biotech, Germany) and 1% penicillin-streptomycin (Hyclone, USA). The cells were incubated at 37℃ in a humidified chamber containing 5% CO_2_.

### Cell transfection

Small interfering RNA (siRNA) sequences were directly synthesized (GenePharma, Shanghai, China). Guide RNA (gRNA) sequences were synthesized by Sangon Biotech (Shanghai, China). Short hairpin RNA (shRNA) was purchased from Tsingke Biotech (Beijing, China). His/myc-tagged Vκ4-1/Jκ3 or Vκ1-5/Jκ3 and Flag-tagged ETFA or ETFB were cloned into pcDNA3.1 vector. The siRNA, shRNA and gRNA were transfected into cell lines using Lipofectamine 3000 (Invitrogen, USA), the plasmid DNA were transfected into cell lines using Polyethylenimine (Polysciences, USA) following the manufacturer’s instructions. After transfection, cells were incubated at 37 °C for 6 h before replaced by complete medium and cultured for an additional 48 or 60 h before further assays. The knockdown efficiency was verified by western blot analysis. The sequences of siRNA, shRNA and gRNA are listed in Table S1.

### RNA extraction and PCR assay

Total RNA from HCC tissues and cells was extracted using TRIzol reagent (Invitrogen, USA), and cDNA was synthesized from 2 µg of total RNA using the Revert Aid First Strand cDNA Synthesis Kit (Thermo Fisher Scientific, USA) according to the manufacturer’s instructions. To determine the *Igκ* rearrangement pattern in HCC cells, semi-nested touchdown PCR was performed to amplify *IGKV* gene transcript, with annealing temperature from 60 °C to 48 °C. The cDNA library of peripheral blood mononuclear cells (PBMCs) was used as a positive control, and reaction systems without templates as a negative control. To determine the expression of FAO-related genes after silencing Igκ in HCC cells, quantitative real-time PCR analysis was performed with Hieff^®^ qPCR SYBR^®^ Green Master Mix (YESEN, Shanghai, China). The expression of the target genes was normalized to *GAPDH* and determined by the 2^−∆∆Ct^ method. The primers used are shown in Table S2.

### Cell proliferation assays

Cell proliferation and viability were assessed using Cell Counting Kit-8 (CCK-8, Dojindo Molecular Technologies, Japan) based on the manufacturer’s protocol. Transfected cells were seeded in 96-well plates at a density of 2000 cells per well with 100 µl of medium per well. At designated time points (0, 24, 48, 72 and 96 h), 10 µl of CCK-8 solution was added to each well, and the plate was incubated at 37 ℃ for 2 h. The absorbance at 450 nm was then measured using a microplate reader and recorded.

For colony formation assays, transfected cells were seeded in 12-well plates at 400 cells per well and incubated for 7–10 days. When visible clones appeared, 1% crystal violet was added to the stain at room temperature for 20 min, and the number of clones were counted.

### Cell migration assays

For wound-healing assays, transfected cells were seeded in 6-well plates until confluence, and a straight line was scraped using a 200 µl pipette tip. The plate was imaged at the same position and at indicated time points under a light microscope. The relative cell migration rate was analyzed using ImageJ software.

For transwell assays, 250 µl of serum-free medium containing 1.5 × 10^5^ transfected cells were seeded in the upper chambers with 8 μm pore (Corning, USA), and 750 µl complete medium containing 10% FBS was added to the bottom of 24-well plates as a chemoattractant. The cells were maintained at 37℃ for 48 h, and the upper chambers were then removed. The migrated cells on the lower surface of upper chambers were stained with crystal violet and quantified using ImageJ software.

### Cell apoptosis detection

Transfected cells were harvested and washed in ice-cold PBS. Suspended cells were then detected in binding buffer with Annexin V-FITC for 15 min and subsequently stained with 7-AAD for 5 min in the dark at room temperature. Cells were detected with a BD FACS Verse instrument (BD Biosciences, USA), and the results were analyzed by FlowJo-V10 software.

### Animal studies

The *Igκ*^*fl/fl*^ mice were mated with *Albumin-cre* transgenic mice to generate *Alb-cre*^*−*^: *Igκ*^*fl/fl*^ (wild-type, WT) mice and *Alb-cre*^*+*^: *Igκ*^*fl/fl*^ (knockout, KO) mice. All experimental procedures were approved by Peking University Laboratory Animal Research Committee and adhered to the Institutional Animal Care and Use Committee of China.

For the HCC mouse model induced by DEN and CCl4, 2-week-old male mice were intraperitoneally injected with 25 mg/kg DEN. At 4 weeks of age, the mice were injected intraperitoneally with 20% CCl4 (5 mg/kg) in corn oil twice a week until 26 weeks. The mice were sacrificed at indicated time point, and the sera were collected for detection. The morphology of mice liver tissue was observed using immunohistochemistry (IHC), hematoxylin and eosin (H&E) and Masson staining.

For the xenograft tumor formation in vivo, the 5 × 10^6^ Huh7 cells transfected with Igκ shRNA or control shRNA in 150 µl of PBS containing 50 µl of Matrigel (Corning, USA) were injected subcutaneously into the flanks of BALB/c nude mice. Tumor growth was monitored every 8 days for the first 38 days and every 4 days thereafter, and the tumor volume was calculated as (width^2^ × length)/2.

### Serum biochemistry

Blood was collected from HCC mouse model, and the serum level of alanine aminotransferase (ALT) and aspartate aminotransferase (AST) was measured using standard enzymatic procedures according to the manufacturer’s instructions (C009-2-1, C010-2-1, Nanjing Jiancheng Bioengineering Institute, Nanjing, China).

### Histology and immunohistochemistry (IHC)

The liver pathology of HCC mouse model was examined. Liver tissues were fixed in 10% neutral-buffered formalin and embedded in paraffin. Tissue sections were stained with H&E and Masson (D026-1, Nanjing Jiancheng Bioengineering Institute, Nanjing, China) according to the manufacturer’s instructions to observe the level of tissue damage using light microscopy.

Liver tissues of human and mouse were subjected to immunohistochemistry. Tissue sections were deparaffinized in xylene, dehydrated in an ethanol gradient, and subjected to antigen retrieval in 10 mM citrate buffer (pH 6.0) in a heater. To inactivate endogenous peroxidase activity, the sections were treated using a 3% peroxidase solution for 10 min and then blocked with goat serum at 37 °C for 1 h. The sections were incubated with indicated antibodies at 4 °C overnight, followed by the incubation with HRP-conjugated anti-mouse/rabbit IgG at RT for 30 min. The immunoreactivity was visualized using an enhanced diaminobenzidine kit (Dako, Denmark) and nuclear staining with hematoxylin. The expression level was quantified using a four-tier intensity score (0, none; 1, weak; 2, moderate; 3, strong) and the percentage (0-100%) of positive cells. The final staining score was obtained by multiplying the scores for the intensity and percentage of positive cells (range, 0-300). Scores *>* 100 were defined as high, while scores ≤ 100 were defined as low.

### Western blot analysis

Total proteins were extracted from HCC tissues or cell lines using RIPA buffer (Beyotime, Shanghai, China) with a complete protease inhibitor cocktail and phosphatase inhibitors (Roche, Basel, Switzerland). Aliquots of protein extracts were separated on 12% SDS-PAGE and transferred to nitrocellulose membranes. The membranes were blocked with 5% milk for 1 h at room temperature and incubated with primary antibodies overnight at 4 ℃. After washing with TBST, membranes were incubated with peroxidase-coupled secondary antibodies for 1 h at room temperature. Protein bands were visualized using an ECL kit (Thermo Fisher Scientific, USA) and iBright™ CL750 imager. The antibodies used in this assay are listed in Table S3.

### Co-immunoprecipitation (Co‑IP) assay and mass spectrometry

Huh7 cell lysates were prepared in lysis buffer (50 mM Tris, 150 mM NaCl, 1% NP-40, pH 7.5), and incubated with anti-Vκ4-1/Jκ3 antibody or isotype control overnight at 4 ℃ with rotation, followed by immunoprecipitation with protein G beads at 4 ℃ for 4 h. After extensive washing to remove the non-specific binding proteins, precipitates were examined by western blot analysis using the indicated antibodies. Mass spectrometry was performed at the Institute of Biotechnology of Peking University Health Science Center (Beijing, China).

For Co-IP assays, protein lysates were prepared from HEK 293T cells in a buffer containing 50 mM Tris, 150 mM NaCl, 1% NP-40 (pH 7.5), and a mixture of protease and phosphatase inhibitors. The lysates were then incubated with anti-His-tag mAb-Magnetic Beads (MBL, Japan) or anti-Flag-tag mAb-Magnetic Beads (Abmart, Shanghai, China) at 4 ℃ for 2 h. After washing with wash buffer (50 mM Tris, 150 mM NaCl, 0.1% NP-40), the precipitates were analyzed by western blot analysis.

### Immunofluorescence (IF) staining

Slides were seeded in the bottom of 24-well plate, and when cell confluence reached 70–80%, the medium was discarded and the cells were gently washed with PBS three times. To determine the cell localization of ETFA, Mito-Tracker Red CMXPos (Beyotime, Shanghai, China) was used as a positive control for mitochondrial staining. The pre-heated Mito-Tracker Red CMXRos solution (200 nM) was added to the cells and incubated at 37℃ for 30 min. The cells were then fixed with 4% paraformaldehyde and permeabilized with 0.5% Triton X-100. After blocking with 10% goat serum, the cells were incubated with primary antibody overnight at 4 ℃. After washing with PBS three times, the fluorescent secondary antibodies were added, and the samples were incubated for 1 h at room temperature in the dark. Finally, the cells were mounted with a DAPI-containing mounting medium that resists fluorescence quenching (Vector Labs, USA), and images were observed and collected using a confocal microscope with an Olympus FV3000 imager.

### RNA sequencing

Total RNA from control or Igκ knockdown Huh7 cells was isolated for RNA sequencing (RNA-seq), which was performed by Novogene Company (Beijing, China). Differentially expressed genes were analyzed using the cloud platform of Novogene. A *P* value < 0.05 and log2 fold change > 1 were considered to meet the screening criterion, and the differential genes were enriched by KEGG analysis.

### Label-free proteomics analysis

Total protein was extracted from transfected Huh7 cells and subjected to LC-MS/MS analysis at the Institute of Biotechnology of Peking University Health Science Center (Beijing, China). Label-free quantification (LFQ) was used to compare the abundance of proteins between the Igκ-knockdown and control groups, with GAPDH used to normalize each sample’s LFQ. Significantly altered proteins (*p* value < 0.05 and log2 fold change > 1.5) were subjected to GO-BP (biological process) analysis using the website of https://metascape.org/gp/index.html#/main/step1. Differential proteins are shown by a heat map using R software.

### Untargeted metabolomic analysis

Transfected Huh7 cells were collected and stored at -80 ℃ before UPLC-Q-TOF/MS analysis at Igenecode Technology Company (Beijing, China). For LC-MS analysis, samples were treated with 1 mL of cold methanol/acetonitrile/H_2_O (2:2:1, v/v/v) and centrifuged at 13,000 rpm and 4 ℃ for 15 min to obtain supernatants. The supernatants were mixed with 100 mL acetonitrile/ H_2_O (1:1, v/v). Analyses were performed using a UHPLC system (1290 Infinity LC, Agilent Technologies, USA) coupled to a quadrupole time-of-flight instrument (AB Sciex TripleTOF 6600). The stability and repeatability of the instrument analysis were monitored using quality control (QC) samples.

### Metabolite measurement

Transfected Huh7 cells seeded in 10 cm dishes were grown to 80% confluence and used for the measurements of various metabolites. Cells were treated with n-Heptane: isopropanol (1:1) for triglyceride measurement, n-Heptane: absolute methanol: chloroform (24:1:25) for free fatty acid measurement or extracting solution for ATP measurement, and then sonicated. After centrifugation, the supernatant was used to determine triglyceride level using a Triglyceride Levels Assay Kit (BC0652), Free Fatty Acid Levels Assay Kit (BC0595) or an ATP Levels Assay Kit (BC0305) purchased from Beijing Solarbio Science & Technology Co. LTD (Beijing, China) according to manufacturer’s instructions.

### Oil Red O staining assay

Oil Red O (ORO) staining was performed with an Oil Red O Kit (G1262, Beijing Solarbio Science & Technology Co. LTD, China). Cells were washed with PBS twice and fixed with the fixative buffer for 30 min. Then cells were washed with distilled water twice and then incubated in 60% isopropanol for 1 min. The newly prepared Oil Red O staining solution was added and soaked for 20 min. Mayer hematoxylin staining solution was added for 2 min. After washing, the cells were observed and photographed under a light microscope (BZ-X800, Japan).

### BODIPY™ 493/503 staining assay

Cells were fixed with 4% paraformaldehyde, washed with PBS, and stained with 0.2 ng/µl BODIPY™ 493/503 (Thermo Fisher Scientific, USA) at room temperature for 15 min. The cells were then mounted with a DAPI-containing mounting medium to visualize the nuclei, and the images were observed and collected under a fluorescence microscope (Olympis FV3000, Japan).

### Statistical analysis

The data were statistically analyzed and graphed with Prism software version 8 (GraphPad, CA, USA). The differences between two groups were determined using two-tailed *t* tests. One-way ANOVA and two-way ANOVA were used to analysis the differences between univariate and multivariate variables among the three groups of data. Kaplan-Meier and log-rank tests were used to determine survival rates. All statistical tests were two-sided probability tests. Significance was indicated as follows: ****p < 0.0001, ***p < 0.001, **p < 0.01, and *p < 0.05.

## Results

### Igκ, especially Vκ4-1/Jκ3-Igκ, is upregulated and associated with a poor prognosis in HCC

**Fig. 1:**
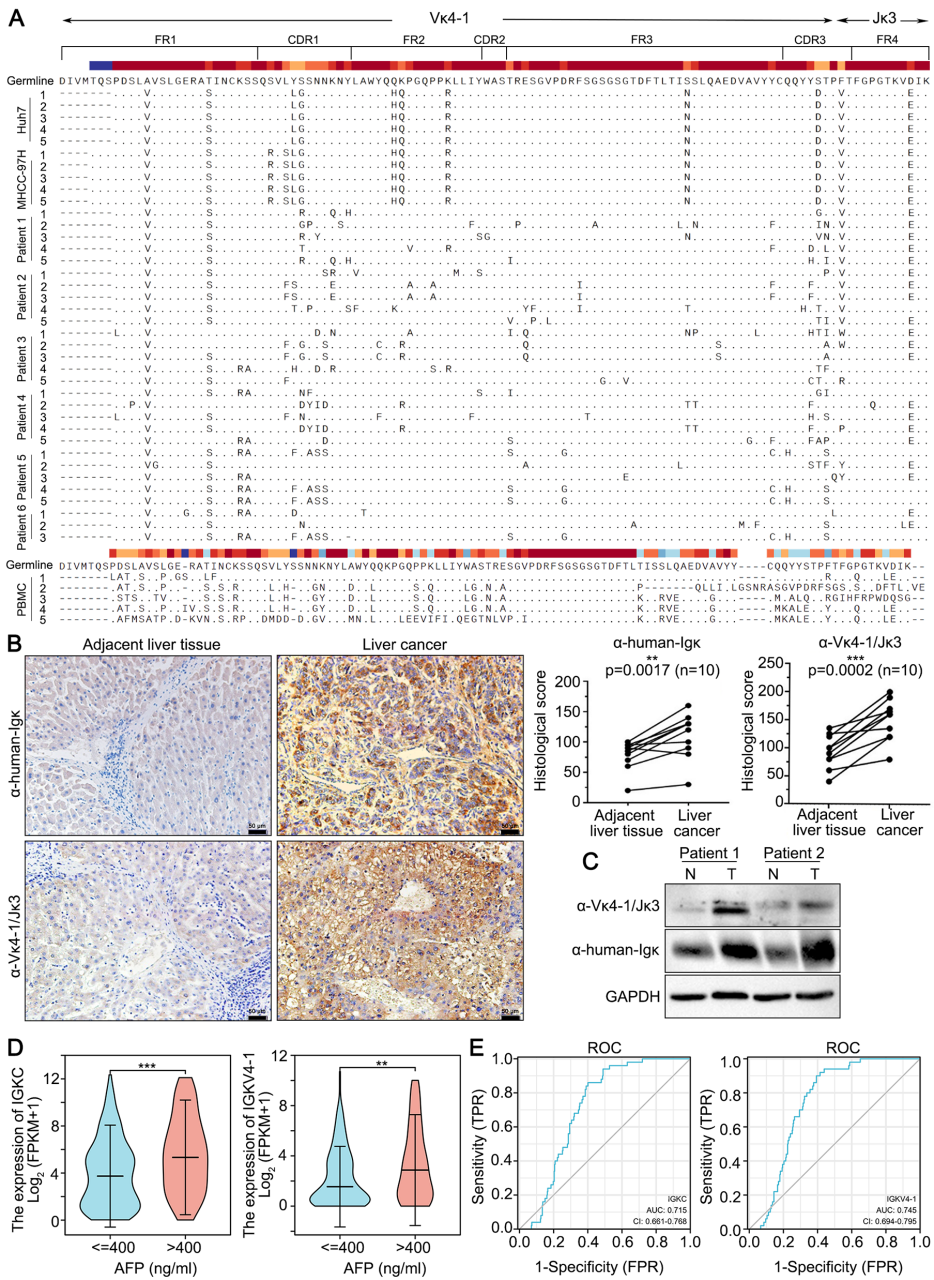
Hepatocyte-derived Igκ is highly expressed in HCC cells and is associated with a poor prognosis. **A** Alignment of Vκ4-1/Jκ3 sequences amplified from HCC cells. Degenerate primers for amplifying all possible variable regions were used in RT-PCR for the detection of *IGK* transcripts in HCC tissues and cell lines. All sequences from HCC cells, including the HCC cell lines Huh7 and MHCC-97 H and 6 HCC tissue samples, are presented; they were aligned with their germline counterparts (marked germline). Peripheral blood mononuclear cell (PBMC) was used as a positive control. **B** Representative images of IHC staining for Igκ and Vκ4-1/Jκ3-Igκ in liver cancer tissues and adjacent liver tissues (left). Statistical analysis was conducted to evaluate the differences between 10 matched cancer tissues and adjacent normal tissues (right) (*n* = 10). Scale bars, 50 μm. **C** The expression of Igκ and Vκ4-1/Jκ3-Igκ was evaluated via western blot analysis in 2 cases of matched HCC tissues and adjacent normal tissues. GAPDH was used as a loading control. **D** The correlation of *IGKC* and *IGKV4-1* with AFP expression. **E** ROC curve analysis of the correlation between *IGKC* or *IGKV4-1* and HCC prognosis. The data are presented as the mean ± SD. ** *p <* 0.01, *** *p <* 0.001

Given that elevated expression of Igκ with a unique Vκ4-1/Jκ3 rearrangement has been confirmed in colon cancer cells [12], we first identified the features of the Igκ light chain in HCC cells. RT-PCR and Sanger sequencing were performed on the cDNA library of two HCC cell lines, Huh7 and MHCC-97 H, and we unexpectedly found that most of the Igκ light chain variable regions exhibited a unique Vκ4-1/Jκ3 rearrangement pattern. We subsequently detected the Vκ/Jκ rearrangement pattern in primary HCC cells from six liver cancer patients; Igκ with the Vκ4-1/Jκ3 rearrangement pattern was verified to be the dominant Igκ light chain in these HCC patients, accounting for 62.5%, 75%, 70%, 77.8%, 62.5% and 42.8% (Fig. 1A and Table S4), which is similar to our previous results in other cancer cells. In addition, *IGK* gene transcripts with different Vκ/Jκ rearrangement patterns were found to be expressed in various human HCC tissues and cell lines through integrative analysis of Gene Expression Omnibus (GEO) datasets (Table S5).

To further identify whether Igκ is highly expressed in HCC, we performed immunohistochemical staining to evaluate the expression patterns of Igκ in 10 pairs of HCC tissues and matched adjacent non-tumor tissues using a commercial anti-human Igκ polyclonal antibody specific for the Igκ constant region and a mouse monoclonal antibody specific for non-B-cell-derived Vκ4-1/Jκ3-Igκ, which is produced with a peptide chain consisting of the first 28 N-terminal amino acids of the Vκ4-1/Jκ3 chain as an immunogen [12]. The results revealed that Igκ was highly expressed in HCC tissues compared with matched adjacent non-tumor liver tissues; specifically, there was significant positive staining of Vκ4-1/Jκ3-Igκ in liver cancer cells but weak positive staining in paracancerous liver cells (Fig. 1B). Next, we utilized the two aforementioned antibodies to assess Igκ expression in both tumor tissues and adjacent non-tumor tissues from HCC patients via western blot analysis. As shown in Fig. 1C, the protein expression level of Vκ4-1/Jκ3-Igκ was significantly greater in the tumor tissues than in the adjacent liver tissues when a commercial anti-human Igκ polyclonal antibody and a specific anti-human Vκ4-1/Jκ3-Igκ monoclonal antibody were used. We next assessed the Igκ staining pattern in two HCC cell lines, Huh7 and MHCC-97 H, by confocal microscopy analysis. Igκ staining of Huh7 and MHCC-97 H cells revealed a cytoplasmic filamentous network with a pointed shape within the nucleus (Fig. S1A). The results of western blot analysis also revealed that Vκ4-1/Jκ3-Igκ was present in both monomeric and polymeric forms in the total cell lysate, whereas in the cell culture supernatant, it was present primarily in a polymeric form under reducing conditions (Fig. S1B). Interestingly, we found that Vκ4-1/Jκ3-Igκ might exist in a free polymer form that does not bind to IgG under nonreducing conditions (Fig. S1C). These findings collectively indicate that Igκ, especially Vκ4-1/Jκ3-Igκ, is highly expressed in HCC cells and may be involved in HCC development.

Furthermore, we used the TCGA database on the XIANTAO platform (http://www.xiantao.love/) to assess the correlation between *IGK* and HCC prognosis. Liver tissues obtained from HCC patients with elevated serum alpha-fetoprotein (AFP) levels (exceeding 400 ng/ml) presented higher expression of *IGKC* and *IGKV4-1* than those from HCC patients with low serum AFP levels (Fig. 1D). Receiver operating characteristic (ROC) curve analysis demonstrated that the diagnostic efficiency of both *IGKC* and *IGKV4-1* exceeded 0.7 (Fig. 1E), suggesting that Igκ is a potential prognostic factor associated with the progression of HCC.

### Hepatocyte-derived Igκ promotes the proliferation and migration of HCC cells

**Fig. 2:**
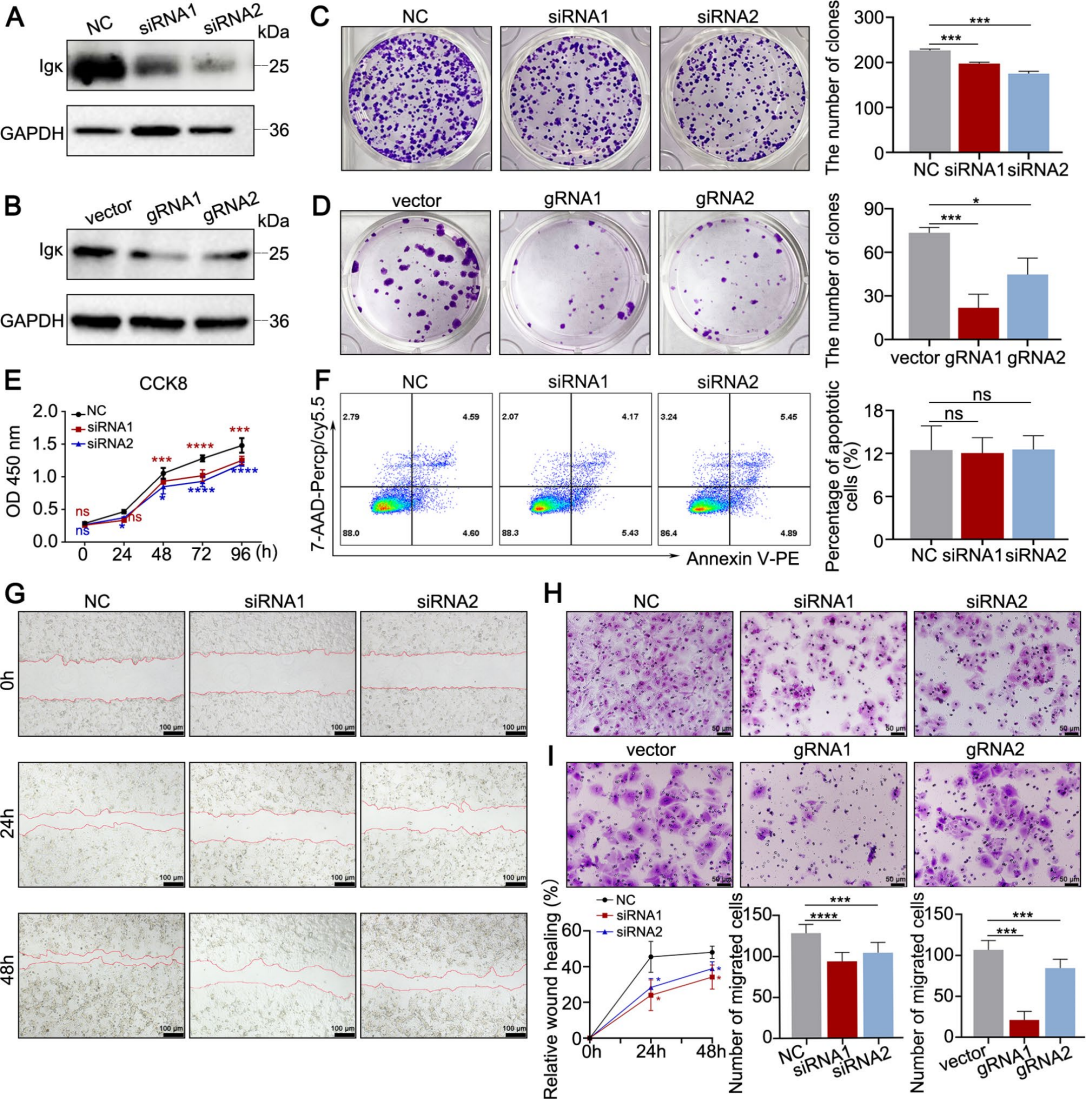
Hepatocyte-derived Igκ promotes the proliferation and migration of HCC cells. **A **- **B** Western blot analysis of Igκ protein levels in Huh7 cells was performed to detect the effect of Igκ knockdown by siRNA or Igκ knockout by the CRISPR-Cas9 system. **C - D** A colony formation assay was conducted to detect the proliferation capacity of Huh7 cells after the knockdown (C) or knockout (D) of Igκ (middle). The quantification of the number of colonies is shown (right) (*n* = 3). **E** A CCK-8 assay was performed to detect the proliferative capacity of Huh7 cells at different time points after Igκ was knocked down (*n* = 3). **F** Representative plots and quantification of apoptotic Huh7 cells after the knockdown of Igκ via flow cytometric analysis (*n* = 3). **G - I** Wound healing assays (**G**) and transwell migration assays (**H **- **I**) were used to detect the migration ability of Huh7 cells in which Igκ was knocked down by using siRNA or knocked out by using Igκ gRNA. The quantification of the relative wound healing area and migrated cell counts are shown (lower right) (*n* = 3). Scale bar, 100 μm (left), 50 μm (right). The data are presented as the mean ± SD. * *p <* 0.05, ** *p <* 0.01, *** *p <* 0.001, **** *p <* 0.0001, and ns, not significant

We previously reported that Vκ4-1/Jκ3-Igκ, an ECM protein, promotes the proliferation, migration and metastasis of colon cancer cells in vitro and in vivo [12]. To explore the biological effects of hepatocyte-derived Igκ, two siRNAs and two guide RNAs (gRNAs) specific for the Igκ constant region were constructed to knockdown or knockout Igκ respectively, and then, efficient knockdown/knockout of Igκ was confirmed (Fig. 2A and B). The results of the colony formation assay in Huh7 cells demonstrated that the number of colonies formed and cell proliferation markedly decreased with Igκ-specific siRNA or gRNA treatment (Fig. 2C and D). Furthermore, a CCK-8 assay demonstrated that Igκ knockdown in Huh7 cells also inhibited cell proliferation in vitro (Fig. 2E). Similar results were obtained for the MHCC-97 H cell line after efficient Igκ knockdown/knockout (Fig. S2A-2E). Since the inhibition of cell proliferation might be due to induced cell apoptosis, we next determined whether Igκ knockdown induces cell apoptosis via Annexin V and 7-AAD staining; no significant difference was detected between the Igκ knockdown group and the control group (Fig. 2F and Fig. S2F). We then aimed to clarify the impact of Igκ on HCC metastasis in vitro. Wound healing assays revealed that a reduction in Igκ expression decreased the wound-healing abilities of Huh7 and MHCC-97 H cells (Fig. 2G and Fig. S2G). Moreover, transwell assay revealed a significant decrease in the number of cells of Igκ-knockdown groups compared with the control groups for Huh7 and MHCC-97 H cells (Fig. 2H and I and Fig. S2H & S2I). Taken together, these results indicate that hepatocyte-derived Igκ plays an important role in HCC cell survival, proliferation and migration.

### Hepatocyte-specific depletion of Igκ alleviates the progression of HCC in mice

**Fig. 3:**
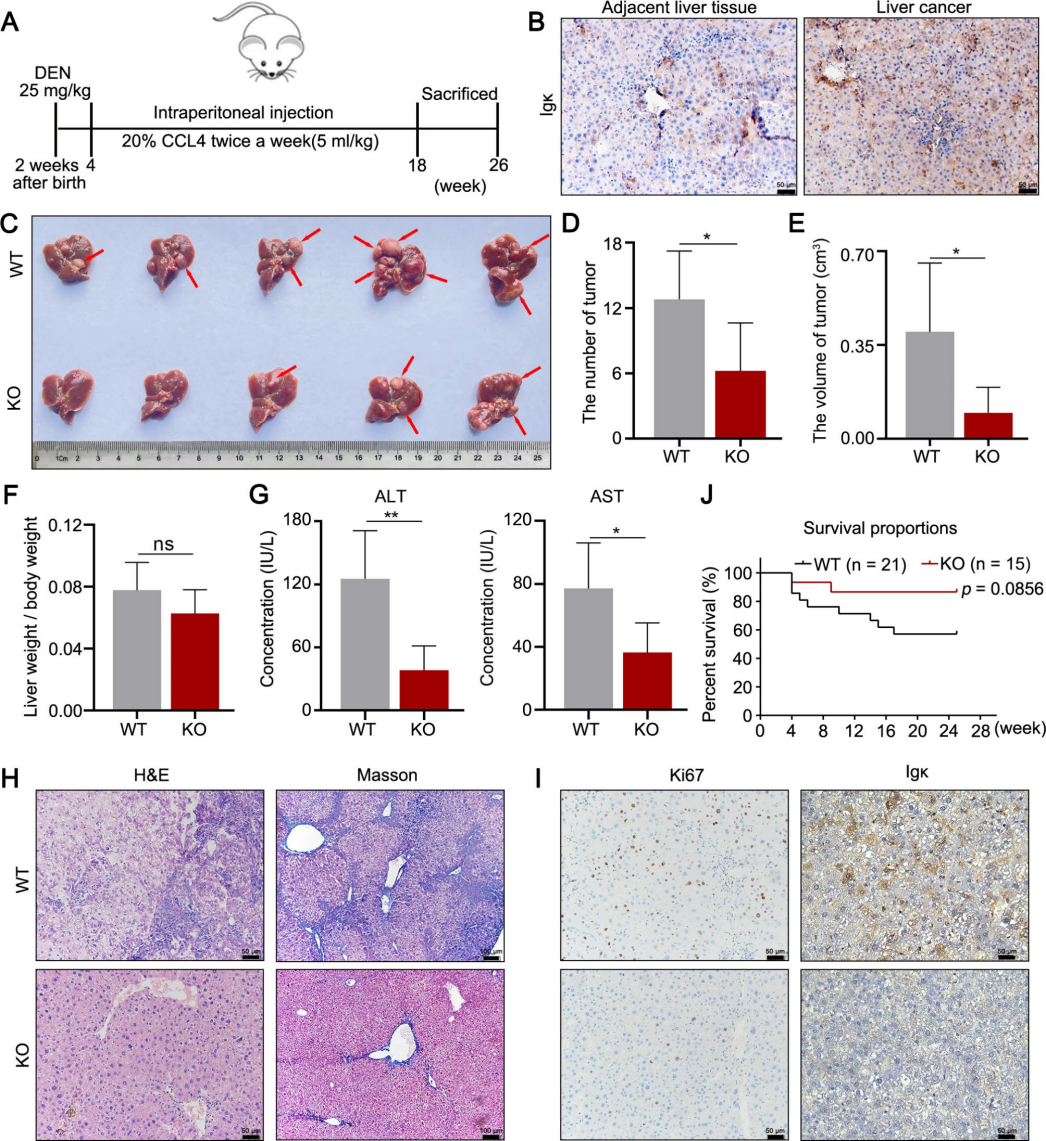
Hepatocyte-specific depletion of Igκ suppresses HCC progression in mice. **A** Schematic representation outlining the procedure for DEN and CCL4-induced HCC mouse models. **B** Representative images of immunohistochemical staining for Igκ protein expression in liver cancer tissues and adjacent liver tissues from mice with HCC. **C** Tumor formation in liver tissues was compared between WT and KO mice, and the red arrow indicates typical tumor nodes (*n* = 5). **D - F** Statistical analysis of the tumor number (D), maximum tumor volume (E) and ratio of liver weight to body weight (F) (*n* = 5). **G** The levels of serum ALT and AST were measured. **H** H&E and Masson staining were performed to assess the pathological changes in the liver tissues of WT and KO mice (*n* = 5). Scale bars, 50 μm and 100 μm. **I** The expression of Ki67 and Igκ in liver tissues was determined by immunohistochemical staining. Scale bar, 50 μm. **J** Survival analysis of WT and KO mice with DEN and CCL4-induced HCC. The data are presented as the mean ± SD. * *p <* 0.05, ** *p <* 0.01

To further investigate the role of hepatocyte-derived Igκ in HCC tumorigenesis in vivo, we generated hepatocyte-specific *Igκ* knockout (*Alb-cre*^*+*^: *Igκ*^*fl/fl*^, KO) mice and control littermates (*Alb-cre*^*−*^: *Igκ*^*fl/fl*^, WT) [15]. Genomic analysis confirmed that treatment with Alb-cre led to the knockout of Igκ (Fig. S3A). Western blot analysis revealed effective depletion of Igκ in hepatocytes from the KO mice compared with those from WT mice (Fig. S3B). WT and KO mice were subsequently treated with DEN (25 mg/kg) at 2 weeks, followed by intraperitoneal injection of 20% CCL4 as a tumor promoter twice a week until 26 weeks to establish an HCC mouse model (Fig. 3A). 100% of the WT mice developed liver tumors at approximately 6 months of age, and tumor status was confirmed via histopathological analysis and Masson staining. Interestingly, as the severity of liver injury and fibrosis in mice gradually increased, Igκ expression increased in liver tissues, as indicated by immunohistochemistry (Fig. S3C). Notably, the positive staining signal for Igκ in tumor tissues was significantly stronger than that in adjacent noncancerous tissues (Fig. 3B). We also found that the number of tumors and maximum tumor volume were significantly lower in the KO mice than in their WT counterparts, even though there was no difference in the liver weight percentage (Fig. 3C and F). Consistently, ALT and AST levels were significantly lower in Igκ-deficient mice than in WT mice (Fig. 3G). Moreover, fewer tumor formation was observed in KO mice than in the WT mice, as determined by H&E staining, and less collagen deposition, as determined by Masson staining, was also in the KO mice (Fig. 3H). IHC staining for Ki67, a proliferation marker closely associated with poor prognosis in clinical conditions [24], revealed that Ki67 expression was markedly decreased in KO mice compared with WT mice, indicating that the hepatocyte-specific depletion of Igκ might suppress liver cancer cell proliferation to alleviate liver tumorigenesis (Fig. 3I). More importantly, the survival rate of the KO mice was greater than that of the WT mice (Fig. 3J). Taken together, these results demonstrated that hepatocyte-derived Igκ deficiency apparently restrains chemically induced hepatocarcinogenesis by downregulating liver cancer cell proliferation.

### Hepatocyte-derived Igκ promotes HCC progression by modulating lipid metabolism

**Fig. 4:**
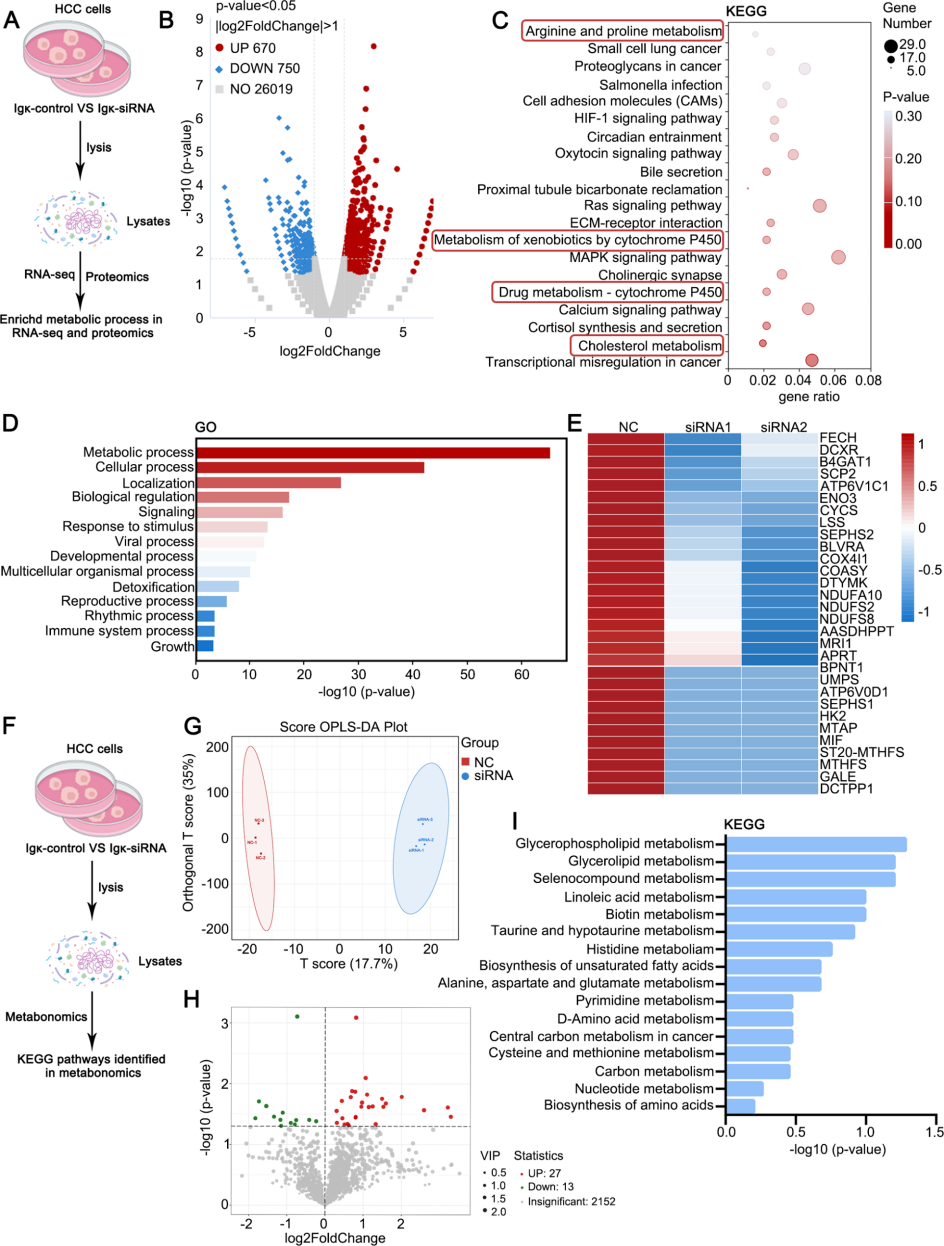
Hepatocyte-derived Igκ promotes HCC progression through the modulation of lipid metabolism. **A** Scheme displaying the procedure used for identifying biological processes regulated by Igκ through RNA-seq and label-free LC-MS/MS proteomic analysis. **B** Volcano plot displaying dysregulated genes (red, upregulated genes; blue, downregulated genes) identified by RNA-seq. **C** The functions of differentially expressed genes were predicted via KEGG enrichment analysis. **D** The functions of differentially expressed proteins in biological processes were subjected via GO enrichment analysis. **E** Heatmap showing differentially expressed metabolism-related proteins upon Igκ knockdown in Huh7 cells. **F** Scheme displaying the procedure used for identifying the specific metabolic process regulated by Igκ through the untargeted metabolomics method. **G** OPLS-DA of metabolomics data was performed to analyse intragroup sample clustering and intergroup differences. **H** Volcano plot displaying dysregulated metabolites (red, upregulated metabolites; blue, downregulated metabolites). **I** KEGG pathway enrichment analysis revealed that glycerophospholipid and glycerolipid metabolism was the most differentially enriched pathway in HCC cells in terms of the number of differentially enriched metabolites

To clarify the mechanism by which hepatocyte-derived Igκ promotes HCC tumorigenesis, we carried out RNA sequencing using total RNA from Huh7 cells transfected with Igκ siRNA or control siRNA (Fig. 4A). A total of 670 upregulated and 750 downregulated genes were detected in the Igκ knockout group compared with the corresponding control group (*p* value < 0.05, log2 fold change > 1.5; Fig. 4B). KEGG enrichment analysis based on all the significantly differentially expressed genes revealed that Igκ knockdown affected multiple metabolic processes, including cholesterol metabolism, drug metabolism-cytochrome P450, and arginine and proline metabolism (Fig. 4C). Moreover, whole-cell lysates of Huh7 cells transfected with Igκ siRNA or control siRNA were extracted for label-free LC‒MS/MS proteomic analysis (Fig. 4A). We identified and quantified 476 proteins, among which 359 were upregulated with a fold change > 1.5 (*p* < 0.05) and 117 were downregulated with a fold change < 1.5 (*p* < 0.05). The differentially expressed proteins in Igκ-knockdown cells were the mostly significantly enriched in the metabolic process according to gene ontology (GO) enrichment analysis (Fig. 4D). The metabolism-related proteins were displayed in a heatmap; among these proteins, cytochrome c, somatic (CYCS) is the central component of the mitochondrial electron transport chain (ETC), cytochrome c oxidase subunit 4I1 (COX4I1) is one of the subunits of ETC complex IV, and NADH: ubiquinone oxidoreductase subunit A10 (NDUFA10), NADH: ubiquinone oxidoreductase core subunit S2 (NDUFS2) and NADH: ubiquinone oxidoreductase core subunit S8 (NDUFS8) encode a subunit of complex I. All of these proteins transfer electrons in the mitochondrial respiratory chain. In addition, other proteins, such as dicarbonyl and L-xylulose reductase (DCXR), enolase 3 (ENO3), hexokinase 2 (HK2) and UDP-galactose-4-epimerase (GALE), are involved in glucose metabolism, whereas beta-1,4-glucuronyltransferase 1 (B4GAT1) and methylthioribose-1-phosphate isomerase 1 (MRI1) play a role in amino acid metabolism. Sterol carrier protein 2 (SCP2) is a peroxisome-associated thiolase involved in the oxidation of branched-chain fatty acids and is closely related to the lipid metabolism pathway (Fig. 4E).

Metabolic reprogramming is fundamental for orchestrating the proliferation and metastasis of tumor cells [21, 25]. Thus, we performed untargeted metabolomics to determine which metabolic pathway is critical for Igκ function (Fig. 4F). On the basis of orthogonal partial least squares-discriminant analysis (OPLS-DA), the metabolomic results revealed that the metabolic profiles were clearly segregated between the Igκ-depletion and control groups, which indicated a consistent and major metabolic shift following the alteration of Igκ expression (Fig. 4G). A volcano plot was subsequently generated to depict the results for 40 metabolites, including 27 upregulated metabolites and 13 downregulated metabolites (*p* value < 0.05, VIP > 1; Fig. 4H). Principal component analysis revealed that Igκ knockdown significantly altered glycerophospholipid and glycerolipid metabolism (Fig. 4I). Therefore, we speculate that hepatocyte-derived Igκ may be involved in HCC development through the regulation of the lipid metabolism pathway.

### Hepatocyte-derived Igκ interacts with ETFA and maintains the stability of ETFA

**Fig. 5:**
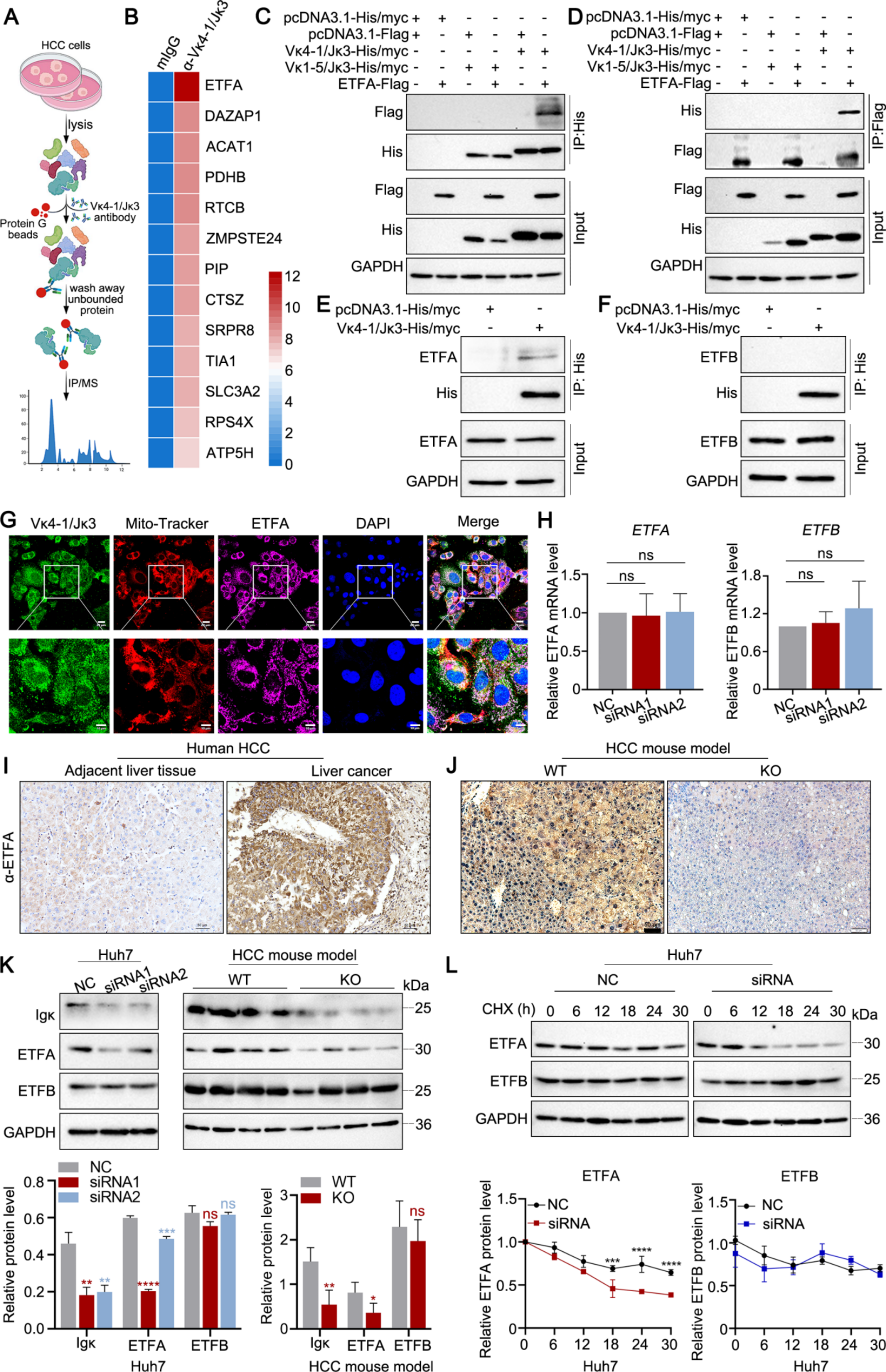
Hepatocyte-derived Igκ interacts with ETFA and maintains the stability of the ETFA protein. **A** Scheme displaying the procedure used for identifying the interacting protein of Igκ through Co-IP assay and mass spectrometry. **B** Heatmap showing the potential binding proteins of Vκ4-1/Jκ3-Igκ as determined by mass spectrometry. **C - D** The 293T cells were transfected with the indicated plasmids, and cell lysates were subjected to Co-IP using anti-His antibody magnetic beads (**C**) and anti-Flag antibody magnetic beads (D). **E - F** Total cell lysates from Huh7 cells overexpressing Vκ4-1/Jκ3-His/Myc were immunoprecipitated with anti-His antibody magnetic beads to detect the interaction between Vκ4-1/Jκ3-Igκ and endogenous ETFA (**E**) and ETFB (**F**). **G** The colocalization of Vκ4-1/Jκ3-Igκ (green) and ETFA (magenta) in mitochondria labelled with Mito-Tracker (red) in Huh7 cells was analysed by confocal immunofluorescence staining. Scale bars, 20 μm (upper) and 10 μm (lower). **H** The levels of *ETFA* and *ETFB* gene transcripts in Huh7 cells after Igκ knockdown were determined by qRT-PCR (*n* = 3). **I** Representative images of IHC for ETFA expression in liver cancer tissues and adjacent liver tissues (*n* = 9). Scale bars, 50 μm. **J** Representative IHC images of ETFA expression in liver tissues from WT and KO HCC mouse models (*n* = 5). Scale bars, 50 μm. **K** The protein levels of ETFA and ETFB in Huh7 cells with Igκ knockdown and liver tissues from WT and KO mice induced with DEN and CCL4 were detected by western blot analysis. Quantification of ETFA and ETFB protein levels relative to control GAPDH (Huh7: *n* = 3, HCC mouse model: *n* = 4). **L** Huh7 cells were transfected with Igκ siRNA or control siRNA and then treated with CHX (50 µg/mL) at the indicated time points. Western blot analysis was performed to evaluate ETFA and ETFB protein levels. The quantification of ETFA and ETFB expression levels is summarized (lower panel) (*n* = 3). The data are presented as the mean ± SD. * *p <* 0.05, ** *p <* 0.01, *** *p <* 0.001, **** *p <* 0.0001, ns, not significant

Next, we aimed to investigate whether Igκ directly regulates lipid metabolism to promote HCC progression; thus, we screened Igκ interactors via Co-IP. The total cell lysate from Huh7 cells was incubated with an anti-Vκ4-1/Jκ3 mouse monoclonal antibody (mAb) or mouse IgG as an isotype control, followed by immunoprecipitation with protein G beads (Fig. 5A). The 13 potential interaction partners for Vκ4-1/Jκ3-Igκ were identified via LC‒MS/MS analysis (Fig. 5B). Among them, ETFA was identified as the most abundant protein, with a peptide score reaching 44.577 (Fig. 5B and Table S6). ETFA is one of the subunits of ETF, and together with electron transfer flavoprotein subunit β (ETFB), to form the ETF complex, which is the third-ranked electron provider in the mitochondrial electron transport chain after complex I and complex II taking up electrons as a hub from at least 14 flavoenzymes and feeding them in the respiratory chain to reduce power for the electron transport chain from FAO and amino acid degradation [26]. Mutations in the *ETFA* genes cause metabolic disorders such as multiple acyl-CoA dehydrogenase deficiency (MADD) [27]. We subsequently investigated whether there is a stable association between ETFA and hepatocyte-derived Igκ. Since Igκ with the Vκ4-1/Jκ3 rearrangement pattern was the dominant Igκ light chain in HCC cells, the plasmids of His-tagged Vκ4-1/Jκ3-Igκ and Vκ1-5/Jκ3-Igκ as a negative control were constructed and overexpressed with Flag-tagged ETFA in 293T cells to perform a co-immunoprecipitation assay. As shown in Fig. 5C and D, Vκ4-1/Jκ3-Igκ but not Vκ1-5/Jκ3-Igκ interacted with ETFA. Moreover, we also confirmed the interaction of Vκ4-1/Jκ3-Igκ and endogenous ETFA in Huh7 cells via a coimmunoprecipitation assay (Fig. 5E), but there was no interaction between Vκ4-1/Jκ3-Igκ and endogenous ETFB in Huh7 cells (Fig. 5F). Confocal immunofluorescence staining further demonstrated that ETFA colocalized with the mitochondrial tracer, which is Mito-Tracker Red CMXPos, and that Vκ4-1/Jκ3-Igκ colocalized with ETFA in the mitochondria of Huh7 cells (Fig. 5G).

To determine whether the interaction between Igκ and ETFA affects their function, we assessed ETFA and ETFB expression levels after Igκ knockdown, and the results of qPCR revealed that Igκ knockdown did not alter ETFA and ETFB transcript levels in Huh7 cells (Fig. 5H). To further clarify whether Igκ regulates ETFA expression at the protein level, we first detected ETFA expression in HCC tissues. Our previous findings indicated that Igκ was highly expressed in HCC tissues compared with the corresponding non-tumor liver tissues, as depicted in Fig. 1B. Consistently, we observed increased expression of ETFA in HCC tissues relative to adjacent tissues (Fig. 5I). Furthermore, ETFA expression was greater in the WT mice than in the KO mice in the HCC mouse model (Fig. 5J). In addition, the protein level of ETFA was obviously decreased in Huh7 cells following siRNA-mediated Igκ knockdown, as determined by western blot analysis and the deletion of Igκ led to a decrease in ETFA protein levels within liver cancer tissues in an HCC mouse model. Moreover, we found that there was no significant change in ETFB after Igκ knockdown in either Huh7 cells or the HCC mouse model (Fig. 5K). Furthermore, we detected the role of Igκ in ETFA protein stability via a cycloheximide (CHX) chase assay. As shown in Fig. 5L, Igκ knockdown resulted in the significant acceleration of ETFA decay but not ETFB decay, indicating that Igκ is likely to reduce ETFA protein stability *via* a post-transcriptional mechanism. Collectively, these findings indicate that Vκ4-1/Jκ3-Igκ interacts with ETFA and further increases ETFA protein stability to participate in the lipid metabolism process.

### Depletion of hepatocyte-derived Igκ disrupts mitochondrial respiration and inhibits fatty acid β-oxidation

**Fig. 6:**
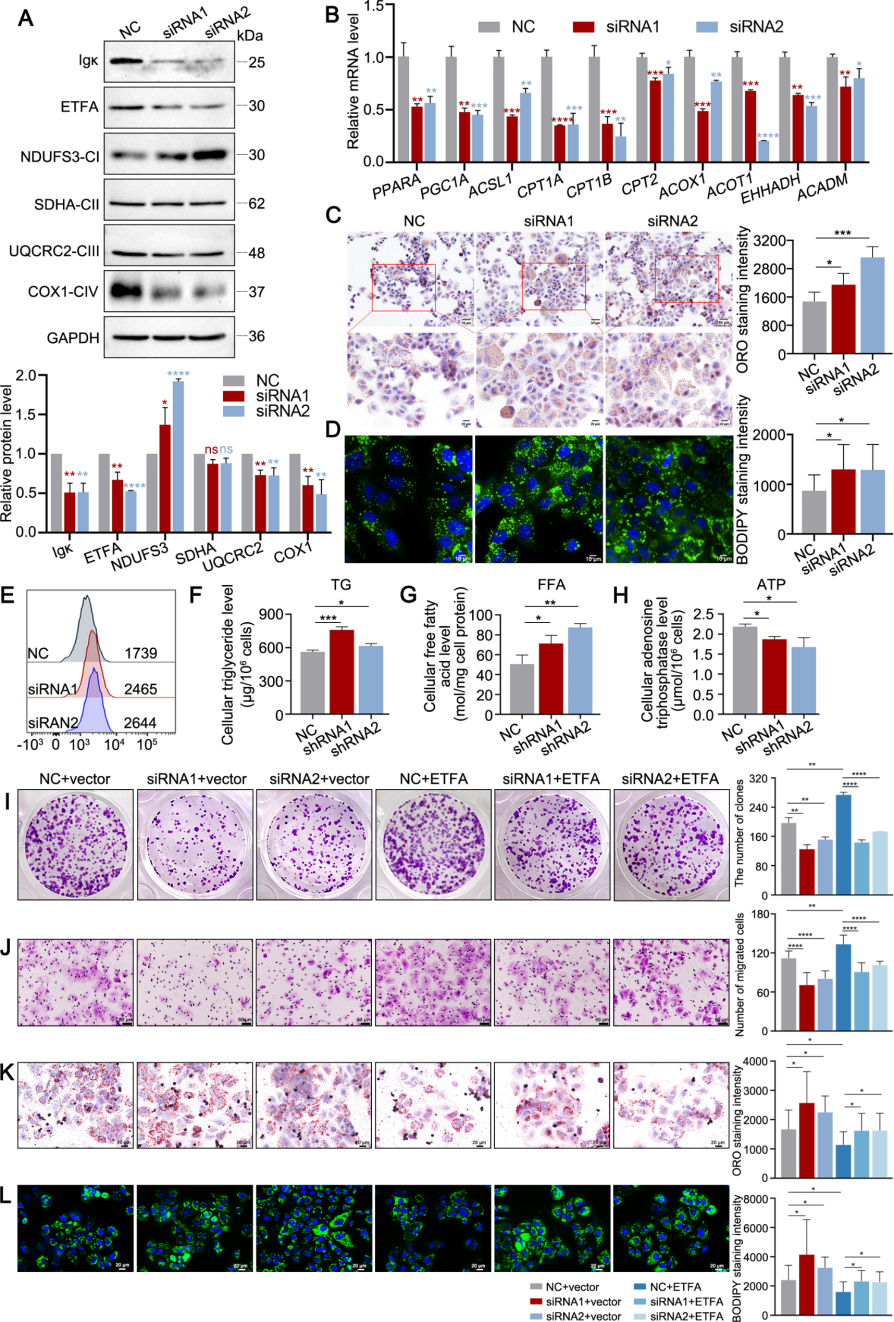
Loss of hepatocyte-derived Igκ impairs mitochondrial respiration and fatty acid β-oxidation. **A** The levels of ETFA, the complex I subunit NDUFS3, the complex II subunit SDHA, the complex III subunit UQCRC2 and the complex IV subunit COX1 after Igκ knockdown in Huh7 cells (left) were detected via western blot analysis. The quantification of the immunoblotting results is shown (right) (*n* = 3). **B** The level of FAO-related gene transcripts in Huh7 cells after Igκ knockdown was determined via qRT-PCR (*n* = 3). **C** Representative images of Oil Red O staining in Huh7 cells after Igκ knockdown. The quantification results for ORO staining intensity are shown (right) (*n* = 3). Scale bars, 50 μm (upper) and 20 μm (lower). **D - E** BODIPY™ 493/503 staining after Igκ knockdown in Huh7 cells was performed, and the fluorescence intensity was analysed by fluorescence microscopy (D) or flow cytometry (E). Scale bar, 10 μm. **F - H** The cellular levels of triglycerides (TG), free fatty acids (FFA) and adenosine triphosphate (ATP) were evaluated in Huh7 cells after treatment with Igκ shRNA (*n* = 3). **I - J** Huh7 cells treated with siRNA against Igκ and pcDNA3.1-vector or pcDNA3.1-ETFA were subjected to colony formation and transwell assays. The quantification results for the number of colonies and migrated cells are shown (right) (*n* = 3). Scale bar, 50 μm. **K** Representative images of Oil Red O staining in Huh7 cells after Igκ knockdown and then transfection with pcDNA3.1-vector or pcDNA3.1-ETFA. The quantification results for ORO staining intensity are shown (right) (*n* = 3). Scale bar, 20 μm. **L** Huh7 cells treated with siRNA against Igκ and pcDNA3.1-vector or pcDNA3.1-ETFA were subjected to BODIPY™ 493/503 staining, and the fluorescence intensity was analysed via fluorescence microscopy. The quantification results for BODIPY™ 493/503 staining intensity are shown (right) (*n* = 3). Scale bar, 10 μm. The data are presented as the mean ± SD. * *p <* 0.05, ** *p <* 0.01, *** *p <* 0.001, **** *p <* 0.0001, ns, not significant

The ETC is composed of four respiratory complexes, including complex I, II, III and IV, which are responsible for electron transfer and generation of the proton gradient across the mitochondrial inner membrane [28]. ETF is the third major electron provider in the mitochondrial ETC after complex I and complex II [29]; thus, we further investigated the effects of Igκ on mitochondrial respiration via western blot analysis and found that Igκ knockdown decreased the levels of ETFA and the complex III subunit ubiquinol-cytochrome c reductase core protein 2 (UQCRC2), in addition to a notable decrease in the complex IV subunit cytochrome c oxidase I (COXI). No significant changes in the complex II subunit succinate dehydrogenase complex flavoprotein subunit A (SDHA) were observed. However, increased levels of the complex I subunit NADH: ubiquinone oxidoreductase core subunit S3 (NDUFS3) were observed, which may indicate a compensatory response of the respiratory chain (Fig. 6A).

ETF delivers electrons in the mitochondrial ETC to both the ubiquinone pool and acyl-CoA dehydrogenases for FAO [30], and FAO is a multistep catabolic process that allows for the mitochondrial conversion of long-chain fatty acids into acetyl-CoA, which is fully oxidized through the TCA cycle and ETC to produce ATP [31]. Before being shuttled into mitochondria for oxidation, fatty acids are activated to fatty acyl-CoA by fatty acyl CoA synthetase (ACS). Carnitine palmitoyltransferase 1 (CPT1) anchor to the mitochondrial outer membrane and facilitates the entry of long-chain fatty acids into mitochondria by loading fatty acyl groups onto carnitine to support FAO [32]. Carnitine palmitoyltransferase 2 (CPT2) on the matrix side of the inner membrane reconverts acylcarnitine to acyl-CoA [31]. PPARG coactivator 1α (PGC-1α)/peroxisome proliferator activated receptor α (PPARα) are important transcriptional regulators of fatty acid metabolism in tissues with high fatty acid oxidation rates including heart, liver, kidney, and skeletal muscle [33, 34]. The binding of PGC-1α to the PPARα/retinoid X receptor (RXR) heterodimer increases the transcriptional activity of genes involved in fatty acid oxidation, such as CPT1 and CPT2 [35]. In addition, acyl-CoA oxidase 1 (ACOX1), acyl-CoA thioesterase 1 (ACOT1), enoyl-CoA hydratase, 3-hydroxyacyl CoA dehydrogenase (EHHADH) and acyl-CoA dehydrogenase medium chain (ACADM) are key enzymes in FAO. Given that hepatocyte-derived Igκ maintains the stability of ETFA in HCC cells, to elucidate the role of Igκ in ETFA-mediated FAO and lipid metabolism, we first performed qPCR analysis following specific Igκ knockdown. The results revealed that Igκ knockdown significantly decreased the expression of FAO-related genes, including *PPARA*,* PGC1A*,* ACSL1*,* CPT1A*,* CPT1B*,* CPT2*,* ACOX1*,* ACOT1*,* EHHADH* and *ACADM* in HCC cells, suggesting that Igκ deficiency suppresses the FAO process (Fig. 6B).

Next, we also observed greater lipid accumulation in Igκ-knockdown HCC cells than in control cells via Oil Red O and BODIPY™ 493/503 staining (Fig. 6C and D). Similarly, the results of FACS analysis using BODIPY ^TM^ 493/503 revealed greater mean fluorescence intensity in Huh7 cells transfected with specific Igκ siRNA than in those transfected with control siRNA (Fig. 6E). As expected, the Igκ shRNA group presented increased concentrations of cellular triglycerides and free fatty acid, and decreased concentrations of adenosine triphosphate (Fig. 6F-H). These results indicate that the knockdown of hepatocyte-derived Igκ downregulated ETFA expression, which disrupted mitochondrial respiration and inhibited FAO.

### Hepatocyte-derived Igκ depletion retards HCC progression by suppressing fatty acid β-oxidation

As mentioned above, ETFA downregulation induced by the loss of Igκ is associated with lipid accumulation. To clarify how the dysregulation of lipid metabolism affects HCC progression, we determined the roles of ETFA in Igκ-regulated HCC cell proliferation and metastasis and lipid metabolism. The results of the colony formation and transwell assays in Huh7 cells demonstrated that cell proliferation and migration were significantly suppressed upon treatment with Igκ siRNA. Moreover, the inhibition of proliferation and migration induced by Igκ knockdown was counteracted by treatment with pcDNA3.1-ETFA (Fig. 6I and J). It has been proven that Igκ knockdown leads to abnormalities in fatty acid degradation pathways, resulting in lipid accumulation. Here, we also observed that induction of lipid accumulation by Igκ knockdown was reversed by supplementation with ETFA, as evidenced by Oil Red O and BODIPY™ 493/503 staining. These findings suggest that ETFA supplementation can ameliorate the lipid metabolism abnormalities induced by ETFA downregulation following Igκ knockdown (Fig. 6K and L).

To further elucidate the regulatory relationship between ETFA and Igκ, we validated the role of ETFA in HCC progression. Endogenous ETFA expression was clearly knocked down via two specific siRNAs against ETFA (Fig. S4A), and ETFA knockdown resulted in a marked decrease in the proliferative activity of Huh7 cells, as determined by colony formation and CCK-8 assays (Fig. S4B and S4C). Similarly, ETFA knockdown reduced the migration ability of HCC cells in the transwell assay (Fig. S4D). Moreover, our results indicated that compared with NC group, Huh7 cells with overexpression of Vκ4-1/Jκ3-Igκ exhibited significantly increased proliferation and migration. However, supplementation with Vκ4-1/Jκ3-Igκ after ETFA knockdown failed to reverse the suppression of cell proliferation and migration (Fig. S4E and S4F). Additionally, ETFA knockdown caused lipid metabolism abnormalities and increased lipid accumulation, which were not alleviated by Vκ4-1/Jκ3-Igκ supplementation (Fig. S4G and S4H). These findings suggest that Vκ4-1/Jκ3-Igκ might function upstream of ETFA and that restoring Vκ4-1/Jκ3-Igκ expression does not effectively counteract the lipid metabolism abnormalities induced by ETFA knockdown.

**Fig. 7:**
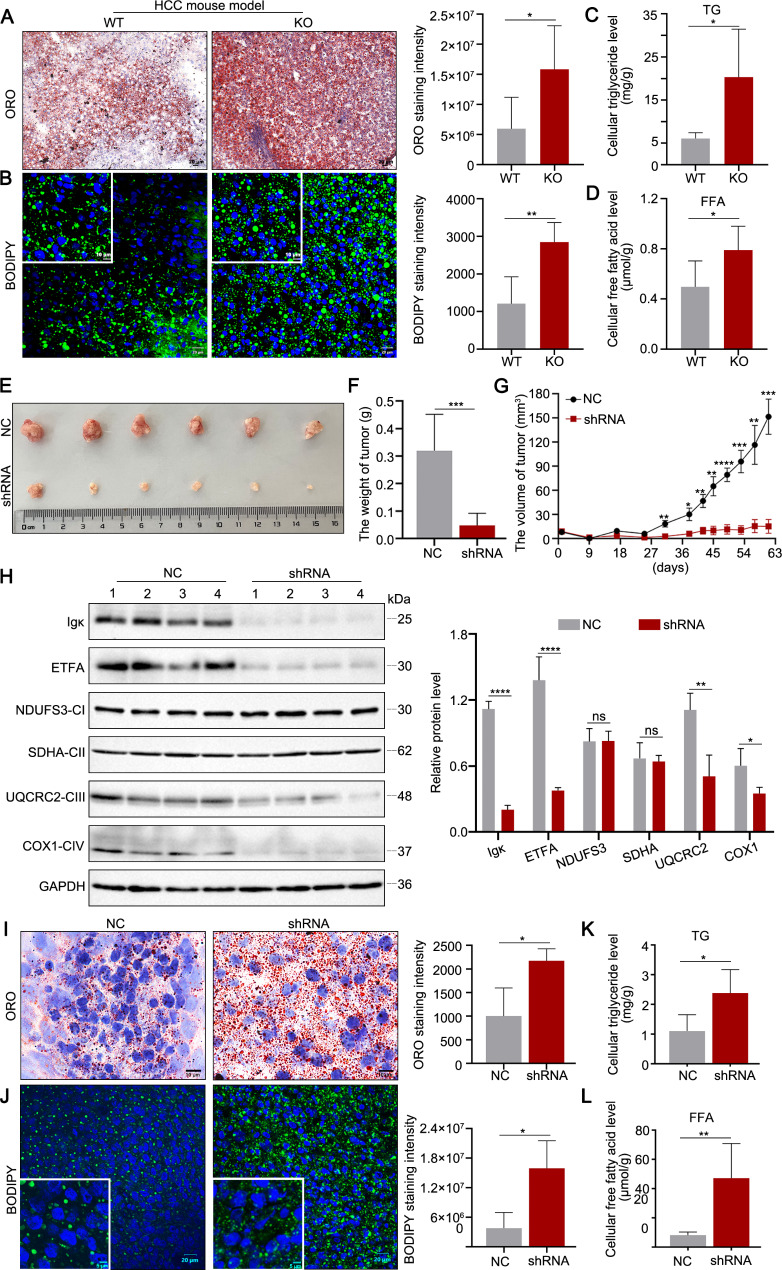
Knockdown of Igκ impairs lipid metabolism and inhibits HCC progression. **A - B** Oil Red O staining and BODIPY™ 493/503 staining were performed on frozen liver tissue sections from WT and KO HCC mouse model (*n* = 5). The quantification results for ORO staining intensity are shown (scale bar, 50 μm), and the fluorescence intensity was analysed via fluorescence microscopy (scale bars, 20 μm and 10 μm). **C - D** The cellular levels of triglyceride (TG) and free fatty acid (FFA) in liver tissues from WT and KO HCC mouse model were evaluated (*n* = 5). **E - F** Huh7 cells transfected with Igκ shRNA or control shRNA (NC) were subcutaneously implanted into nude mice, and tumors were dissected at the end of the experiment. Representative images of tumors (E) and tumor weight data (F) are shown (*n* = 6). **G** Tumour volume was measured every 8 days for the first 38 days and every 4 days thereafter (*n* = 6). **H** Total protein extracted from the xenografts was subjected to western blot analysis and probed with the indicated antibodies. GAPDH was used as a loading control (left). The quantification results for immunoblotting are shown (right) (*n* = 4). **I** Oil Red O staining was performed on frozen tissue sections from the xenografts. The quantification results for ORO staining intensity are shown (right) (*n* = 4). Scale bar, 10 μm. **J** BODIPY™ 493/503 staining of frozen tissue sections from the xenografts was performed, and the fluorescence intensity was analysed via fluorescence microscopy (*n* = 4). Scale bars, 20 μm and 5 μm. **K - L** TG and FFA levels in the xenografts were evaluated (*n* = 4). The data are presented as the mean ± SD. * *p <* 0.05, ** *p <* 0.01, *** *p <* 0.001, **** *p <* 0.0001, ns, not significant

Furthermore, to determine whether Igκ deficiency inhibited lipid metabolism in vivo, we first examined lipid changes in an HCC mouse model. The lipid content was significantly higher in the KO mice than in the WT mice according to Oil Red O and BODIPY™ 493/503 staining (Fig. 7A and B). Moreover, the levels of TG and FFA were greater in the KO mice than in the WT mice (Fig. 7C and D). In addition, we constructed an HCC xenograft mouse model to verify the role of Igκ in HCC lipid metabolism. Huh7 cells with stable Igκ knockdown were established, and these cells were subsequently inoculated into the flanks of nude mice. Tumor growth was monitored every 8 days for the first 38 days and every 4 days thereafter and was visualized via a tumour growth curve. As shown in Fig. 7D–F, significant reductions in tumor volume and weight were detected in the shRNA group compared with the control group. We also investigated the effects of Igκ on mitochondrial respiration via western blot analysis and found that Igκ knockdown decreased the levels of ETFA and the complex III subunit UQCRC2, as well as the complex IV subunit COXI. No significant changes in the complex I subunit NDUFS3 or the complex II subunit SDHA were observed (Fig. 7G). Next, we observed greater lipid accumulation in the Igκ shRNA group than in the NC group via Oil Red O and BODIPY™ 493/503 staining (Fig. 7H and I). Similarly, the levels of TG and FFA in tumor tissue also increased in the shRNA group (Fig. 7J and K). These results demonstrate that hepatocyte-derived Igκ increases ETFA protein stability, thereby stimulating FAO and preventing lipid accumulation, which promotes HCC cell proliferation and migration, exacerbating HCC progression.

## Discussion

**Fig. 8:**
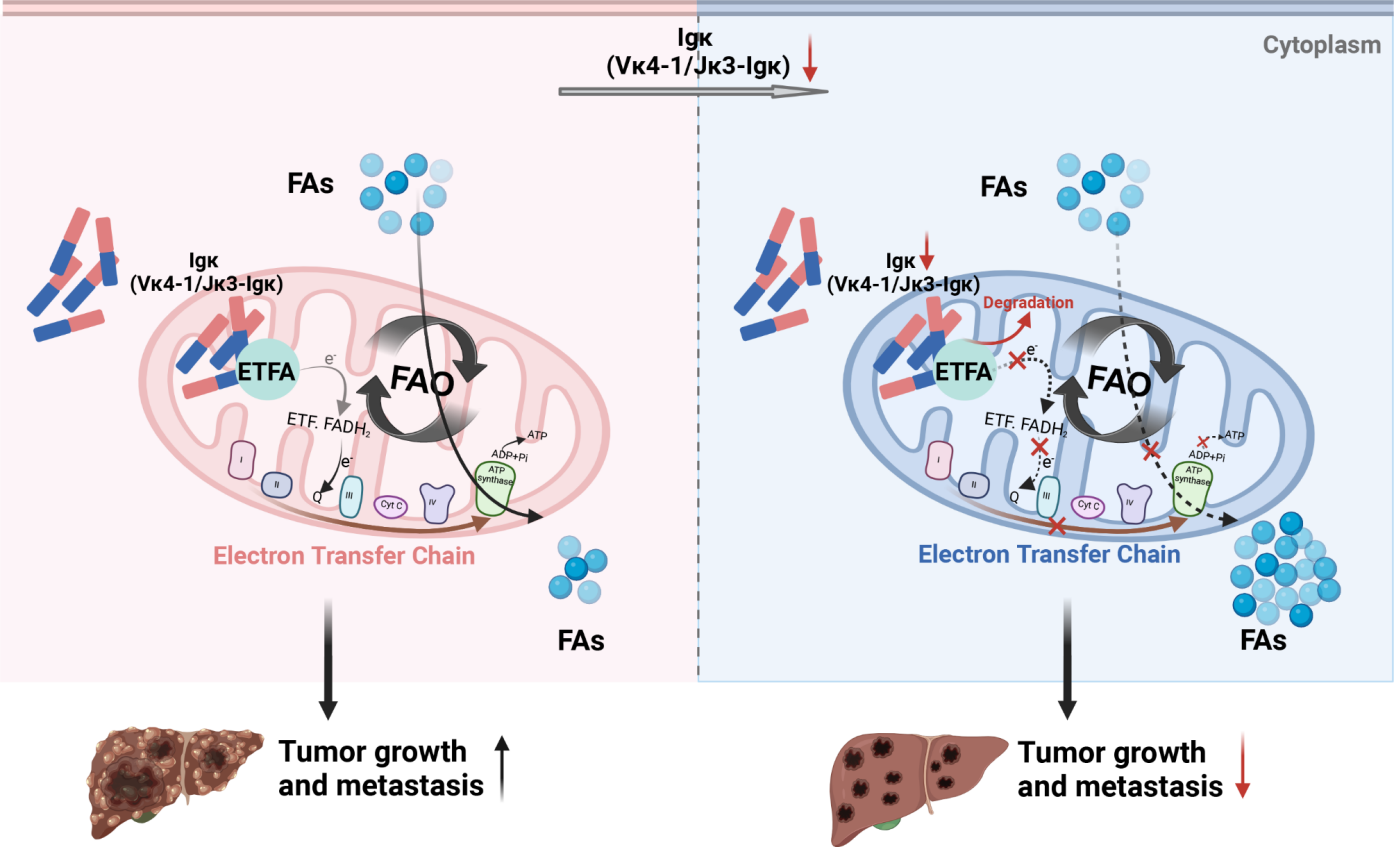
A working model explaining how hepatocyte-derived Igκ promotes HCC progression by stabilizing ETFA to facilitate fatty acid β-oxidation. Hepatocyte-derived Vκ4-1/Jκ3-Igκ interacts with the electron transporter ETFA on the mitochondrial respiratory chain, and loss of Igκ promotes ETFA protein degradation, leading to decreased expression of mitochondrial respiratory chain complexes III and IV, thereby causing aberrant FAO and lipid accumulation. Dysregulated lipid metabolism subsequently inhibits the proliferation and migration of HCC cells, thereby delaying HCC development

Although there is a previous report on the role of non-B-cell-derived Igκ in colon cancer growth and metastasis, its role in HCC tumorigenesis remains unclear. In the present study, we first confirmed that Vκ4-1/Jκ3-Igκ expression was significantly elevated in HCC and verified that hepatocyte-specific Igκ deficiency inhibited HCC progression in a DEN and CCL4-induced HCC mouse model. Interestingly, hepatocyte-derived Igκ interacts with ETFA, an important component of the electron transport chain in mitochondria, and stabilizes its expression to facilitate fatty acid β-oxidation, which is associated with HCC growth and metastasis. Taken together, these results suggest that hepatocyte-derived Igκ modulates lipid metabolic homeostasis by stabilizing ETFA and might be a potential target involved in HCC development (Fig. 8).

Ig is currently considered to be a product of B cells and plasma cells that has antibody activity and a basic four-chain structure consisting of two heavy chains and two light chains [36]. However, increasing evidence has shown that many non-B cells, especially epithelial tumor cells, produce different types of Igs, such as IgG. High levels of IgG derived from tumor cells promote tumor progression, metastasis and drug resistance and are correlated with a poor prognosis [37–41]. More interestingly, we unexpectedly found that various tumor cells highly express Igκ with a unique Vκ4-1/Jκ3 rearrangement pattern, which is homologous to the pathogenic Igκ free light chain (FLC) in LCDD and AL-AM [12]. In addition, tumor-derived Vκ4-1/Jκ3-Igκ has strong hydrophobic characteristics and forms obvious insoluble deposits in the extracellular matrix. Under normal conditions the FLC are present either as monomer or dimer, whereas pathological conditions, somatic mutation or posttranslational modification lead to reorganization of FLC to higher-order polymeric forms that aggregate in local tissue rather than body fluid [42]. Our previous studies revealed that a large number of Igκ were detected in many tissues of B-cell-deficient mice, such as the liver, lung, and kidney [43]. Moreover, Igκ derived from mouse primary hepatocytes exhibited the same restricted rearrangement pattern, and Igκ deposition is significantly linked to ConA-induced liver injury [15]. Our present findings further demonstrated that Igκ with a Vκ4-1/Jκ3 rearrangement pattern was highly expressed in HCC cells in a free polymeric form and that the expression level of Igκ gradually increased with increasing severity of liver injury and fibrosis in mice. Notably, high expression of Vκ4-1/Jκ3-Igκ rearrangement is closely associated with a poor prognosis for HCC. It also has been reported that high expression of Igκ is closely related to a poor prognosis for breast cancer [14]. These findings suggest that high Vκ4-1/Jκ3-Igκ expression in HCC cells might contribute to HCC development.

To further elucidate the role of hepatocyte-derived Igκ, we conducted in vitro experiments to knockdown Igκ in two HCC cell lines and found that the knockout of Igκ significantly inhibited the proliferation and migration of HCC cells. More importantly, we utilized hepatocyte-specific Igκ knockout mice to establish DEN and CCL4-induced HCC models. The specific depletion of Igκ in hepatocytes apparently alleviated HCC development in mice, primarily by suppressing the proliferation of HCC cells. Our previous findings indicated that the Igκ FLC promotes colitis pathogenesis and colitis-associated colon carcinogenesis [44]. Notably, Vκ4-1/Jκ3-Igκ also significantly promotes the proliferation and migration of colon cancer cells both in vitro and in vivo [12]. Although other non-B cells might produce Igκ to perform certain biological functions, the findings of the current study underscore the crucial role of Vκ4-1/Jκ3-Igκ derived from HCC cells in the process of HCC tumorigenesis. Consequently, we hypothesize that hepatocyte-derived Igκ might play an important role in the occurrence and development of HCC.

We further investigated the mechanism of Igκ-mediated HCC tumorigenesis, and the intersection of the results from the proteomics and IP-MS/MS analyses revealed that Vκ4-1/Jκ3-Igκ can interact with ETFA in the mitochondria, and Igκ deficiency led to a decrease in ETFA expression. ETFA forms the complex of electron transfer flavoproteins (ETFs) with ETFB, which are key components of the electron transport chain in mitochondria [26]. ETFs shuttle electrons during one-carbon metabolism to ETF-ubiquinone oxidoreductase (ETF-QO) through its cofactor flavin adenine dinucleotide (FAD) to reduce ubiquinone, which is essential for oxidative phosphorylation in the mitochondrial ETC [26], but also transfers electrons from dehydrogenation reactions of multiple flavoprotein dehydrogenases involved in the β-oxidation process of fatty acids [45]. Therefore, defects in the ETF system prevent the body from metabolizing lipids for energy within the liver and muscles, leading to the accumulation of various intramitochondrial acetyl-CoA esters [46] and ultimately disrupting the lipid metabolism pathway. Mutations in the *Etfa/Etfb/Etf-qo* genes affect the stability of the corresponding proteins, and defects in the ETFA or ETFB protein have been implicated in glutaric aciduria type II (GA-II), a rare autosomal recessive inherited metabolic disorder. In this study, RNA-seq, proteomics and untargeted metabolomics analyses revealed that Igκ knockout significantly altered lipid metabolism in HCC cells. More importantly, we found that loss of Igκ decreased ETFA stability by promoting its degradation in HCC cells, resulting in a reduction in the protein levels of mitochondrial respiratory chain complex III and complex IV, accompanied by downregulation of FAO-related genes at the mRNA level. Owing to dysfunction of the mitochondrial ETC, Igκ deficiency resulted in the accumulation of free fatty acids and triglycerides, as well as a decrease in ATP synthesis through the inhibition of FAO. Although Vκ4-1/Jκ3-Igκ, a potential ECM proteins that bind to integrin β1 by triggering the FAK/Src signalling pathway to participate in colon cancer progression, has been identified [12], no significant change in the FAK/Src signalling pathway was observed in HCC cells after Igκ knockout (data not shown). This discrepancy might be due to the different expression patterns of the Igκ protein, but further study is needed for verification.

Growing evidence indicates that metabolic reprogramming is critically involved in tumor growth and metastasis [47, 48]. In particular, alterations in lipid metabolism resulting from a source of energy, microenvironmental adaptation, and cell signals in neoplastic cells are increasingly recognized for their role as key characteristics during the onset and progression of HCC [49, 50]. In rapidly proliferating HCC cells, carbons are hijacked from energy production to synthesize fatty acids (FAs), which can be sourced either exogenously or from de novo synthesis. FAs stored in triglycerides and sterol esters in the liver can be oxidized to generate ATP via FAO; hence, the FAO process plays a crucial role in the regulation of several oncogenic processes [51, 52]. However, the specific role of FAO in HCC tumorigenesis remains unclear, and this lack of clarity may be due to tumor heterogeneity in HCC. The expression of many β-oxidation-related genes varies significantly among patients [23]. Several studies have indicated that the downregulation of CPT2, which is involved in the FAO process, protects against lipotoxicity in an E2F2-dependent manner in HCC to promote HCC cell proliferation and metastasis [53]. The upregulation of hypoxia inducible factor-1α inhibits β-oxidation, resulting in decreased reactive oxygen species levels and increased glycolysis to further facilitate HCC development [54]. This evidence highlights that targeting β-oxidation is a highly promising strategy for treating HCC. On the other hand, some studies have also indicated that downregulation of the expression of FAO-related genes such as CPT1A and ACSL can reduce the risk of tumor occurrence and metastasis [55, 56]. The induction of liver X receptor α (LXRα) signalling *via* small molecules promotes the synthesis of FFAs, resulting in the accumulation of toxic FAs and ultimately HCC cell death [57]. Our results revealed that the loss of hepatocyte-derived Igκ downregulates the expression of ETFA, causing abnormalities in mitochondrial electron transport to inhibit FAO, accompanied by increases in the levels of cellular TG and FFA, as well as a decrease in ATP, to inhibit the proliferation and metastasis of HCC cells. Thus, targeting Igκ is a potential strategy for inhibiting metabolic reprogramming-driven HCC development and progression.

## Conclusions

In conclusion, this study revealed high expression of hepatocyte-derived Igκ with a unique Vκ4-1/Jκ3 rearrangement pattern and the mode of Igκ regulates FAO by interacting with the electron transporter ETFA in HCC cells. Notably, the loss of Igκ led to an increase in fatty acid availability, which inhibited the proliferation and metastasis of HCC cells. We delineated the role of the novel Igκ/ETFA axis in the regulation of FAO and highlighted the critical function of Igκ in modulating fatty acid metabolism to participate in HCC tumorigenesis. Overall, this study contributes to a better understanding of the mechanistic pathways that shape the dysregulation of fatty acid metabolism in HCC, which will be beneficial for the advancement of targeted therapies for HCC patients.

## Electronic supplementary material

Below is the link to the electronic supplementary material.

Supplementary Material 1

## Data Availability

All publicly available data can be acquired from the corresponding web servers described in the Materials and methods. The data used and/or analyzed in the present study are available from the corresponding author on reasonable request.

## Abbreviations

ACS: Acyl CoA synthetase
ACOX1: Acyl-CoA oxidase 1
ACOT1: Acyl-CoA thioesterase 1
ACADM: Acyl-CoA dehydrogenase medium chain
AL-AM: Amyloid light chain amyloidosis
ALT: Alanine aminotransferase
AST: Aspartate aminotransferase
ATP: Adenosine Triphosphate
CCL4: Carbon tetrachloride
CHX: Cycloheximide
Co-IP: Co-immunoprecipitation
COX4I1: Cytochrome c oxidase subunit 4I1
CPT1: Carnitine palmitoyltransferase 1
CPT2: Carnitine palmitoyltransferase 2
CYCS: Cytochrome c, somatic
DEN: Diethylnirtosamine
DCXR: Dicarbonyl and L-xylulose reductase
EHHADH: Enoyl-CoA hydratase and 3-hydroxyacyl CoA dehydrogenase
ENO3: Enolase 3
ETFA: Electron transfer flavoprotein subunitα
ETFB: Electron transfer flavoprotein subunitβ
ETC: Electron transport chain
FAO: Fatty acid β-oxidation
FLC: Free light chain
FFA: Free fatty acids
GALE: UDP-galactose-4-epimerase
GEO: Gene expression omnibus
HCC: Hepatocellular carcinoma
H&E: Hematoxylin-eosin staining
HK2: Hexokinase 2
Ig: Immunoglobulin
IHC: Immunohistochemistry
IF: Immunofluorescence
LCDD: Light chain deposition disease
LFQ: Label-free quantification
MRI1: Methylthioribose-1-phosphate isomerase 1
MADD: Multiple acyl-CoA dehydrogenase deficiency
NDUFA10: NADH: ubiquinone oxidoreductase subunit A10
NDUFS2: NADH: ubiquinone oxidoreductase core subunit S2
NDUFS8: NADH: ubiquinone oxidoreductase core subunit S8
ORO: Oil red O
PGC-1α: PPARG coactivator 1α
PPARα: peroxisome proliferator activated receptorα
ROC: Receiver Operating Characteristic Curve
SCP2: Sterol carrier protein 2
TCGA: The Cancer Genome Atlas
TG: Triglycerides
