# Supplementary material for: Hepatocyte-derived Igκ promotes HCC progression by stabilizing electron transfer flavoprotein subunit α to facilitate fatty acid β-oxidation
Source: J Exp Clin Cancer Res. 2024 Oct 9;43:280. doi: 10.1186/s13046-024-03203-8 (PMC11462706; doi:10.1186/s13046-024-03203-8)
Supplement: Supplementary file 1 — Supplementary Material 1 [file 13046_2024_3203_MOESM1_ESM.docx]

**Hepatocyte-derived Igκ promotes HCC progression by stabilizing electron transfer flavoprotein subunit α to facilitate fatty acid β-oxidation**

Jingjing Guo^1^, Huining Gu^1^, Sha Yin^1^, Jiongming Yang^1^, Qianqian Wang^1^, Weiyan Xu^1^, Yifan Wang^1^, Shenghua Zhang^1^, Xiaofeng Liu^3^, Xunde Xian^4^, Xiaoyan Qiu^1*^ and Jing Huang^1, 2*^

1 Department of Immunology, School of Basic Medical Sciences, and NHC Key Laboratory of Medical Immunology, Peking University, Beijing, 100191, China

2 PUHSC Primary Immunodeficiency Research Center, Peking University, Beijing, 100191, China

3 Heatopancreatobiliary Surgery Department I, Key Laboratory of Carcinogenesis and Translational Research (Ministry of Education/Beijing), Peking University Cancer Hospital & Institute, Beijing 100142, China

4 Institute of Cardiovascular Sciences, State Key Laboratory of Vascular Homeostasis and Remodeling, School of Basic Medical Sciences, Peking University, Beijing, 100191, China.

*Correspondence:

Xiaoyan Qiu, [qiuxy@bjmu.edu.cn](mailto:qiuxy@bjmu.edu.cn)

Jing Huang, [huangjing82@bjmu.edu.cn](mailto:huangjing82@bjmu.edu.cn)

**Supplementary Figures and Tables:**

**Figure S1**


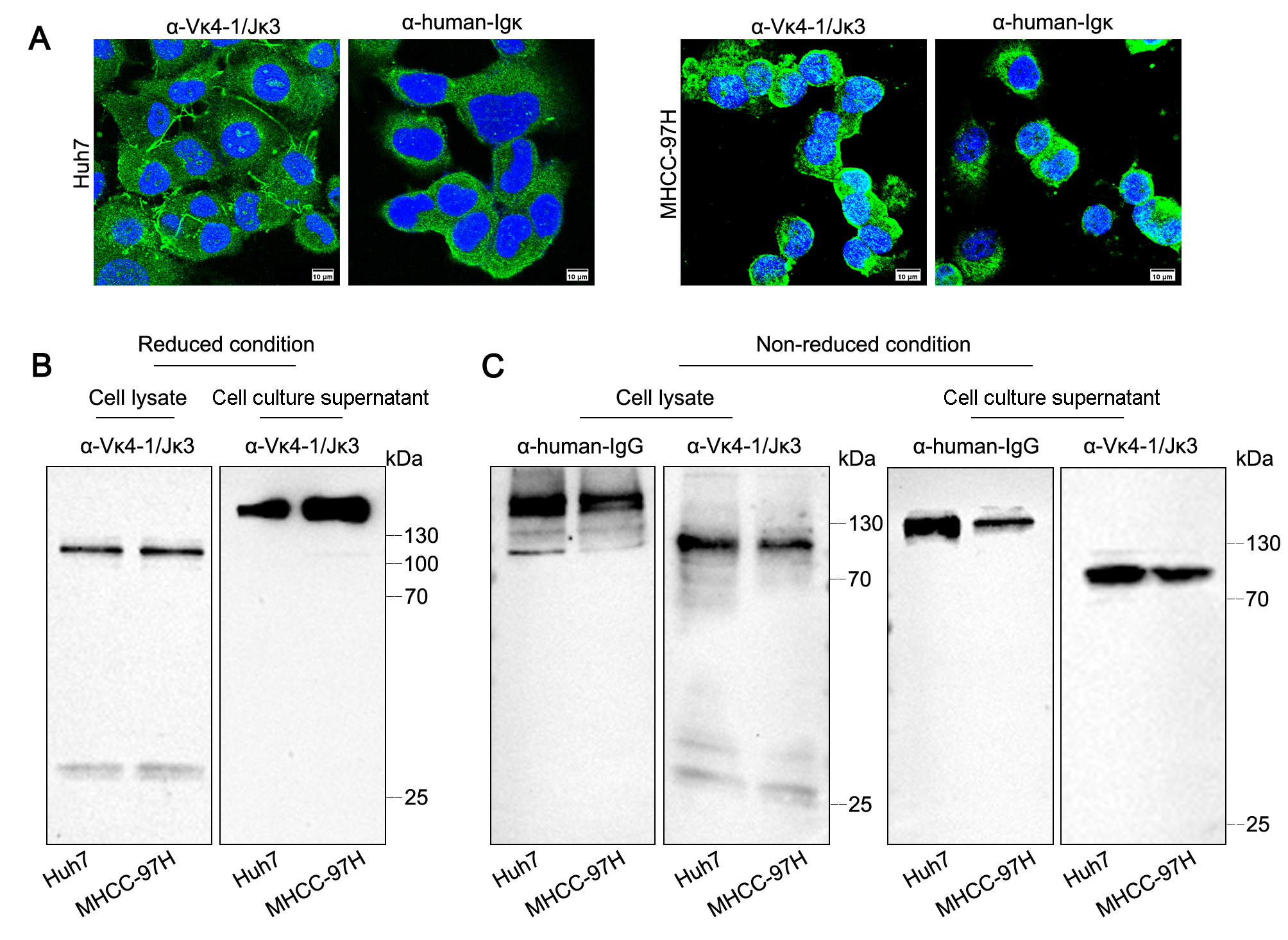


**Fig. S1** **HCC cell lines (Huh7 and MHCC-97H) expressed Igκ.** **A** Immunofluorescence staining of Vκ4-1/Jκ3-Igκ and Igκ with anti-human Vκ4-1/Jκ3 monoclonal antibody and commercial anti-human Igκ in Huh7 and MHCC-97H cells. Green: Vκ4-1/Jκ3-Igκ and Igκ. Blue: DAPI. Scale bar, 10 μm. **B** Expression of Vκ4-1/Jκ3-Igκ in Huh7 and MHCC-97H cell lysate and culture supernatant was detected by western blot under reduced condition. **C** Expression of Vκ4-1/Jκ3-Igκ in Huh7 and MHCC-97H cell lysate and culture supernatant was detected by western blot under non-reduced condition. The expression of IgG as positive control.

**Figure S2**


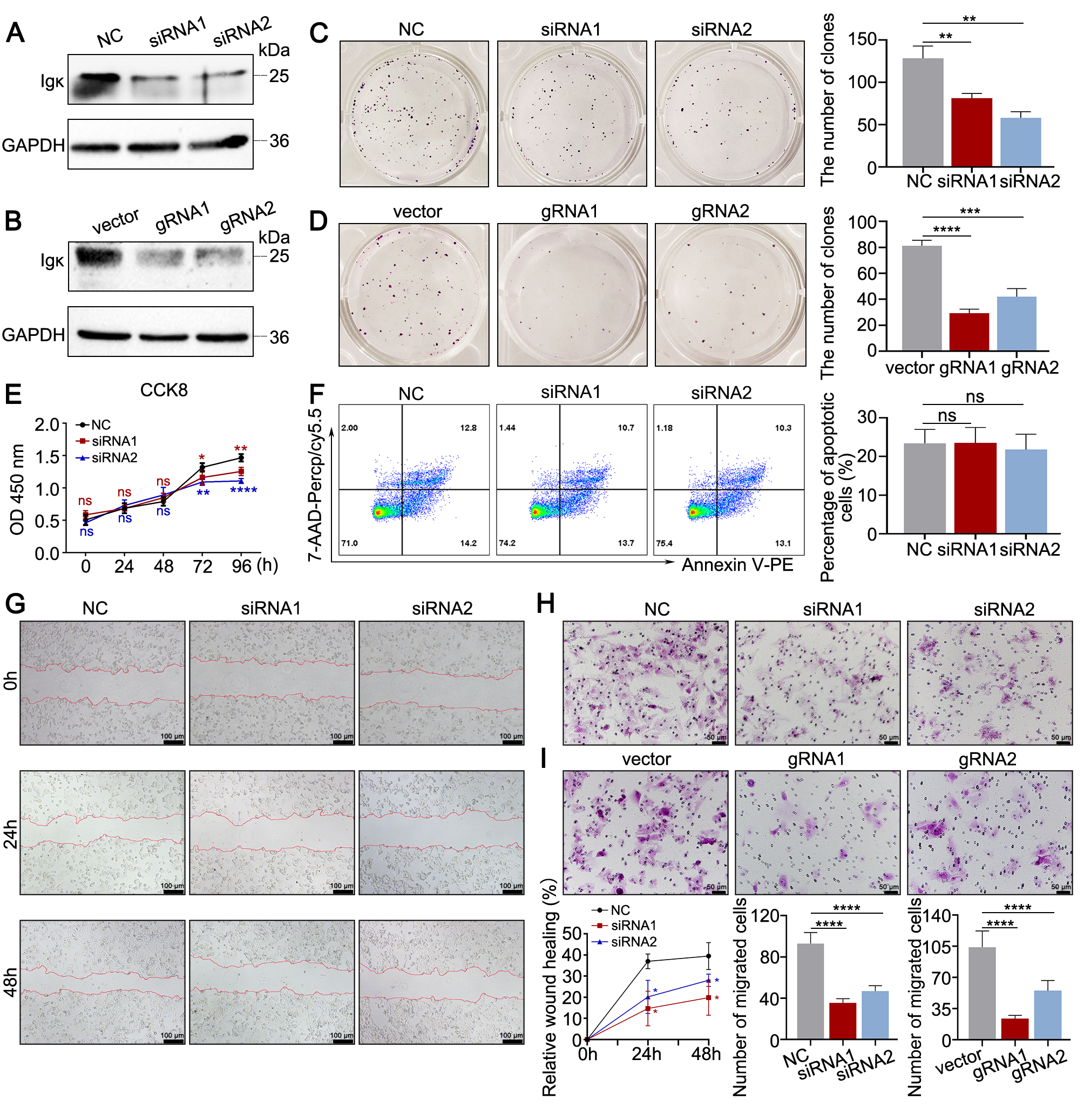


**Fig. S2** **Hepatocyte-derived Igκ promotes proliferation and migration of MHCC-97H HCC cells.** **A - B** Western blot analysis of Igκ protein level in MHCC-97H cells to detect the effect of Igκ knockdown by siRNA or Igκ knockout by CRISPR-Cas9 system. **C - D** The colony formation assay was conducted to detect the proliferation capacity of MHCC-97H cells after knockdown (C) and knockout (D) of Igκ (middle). Quantification of the number of clones are shown (right). **E** CCK-8 assay was performed to detect the proliferation capacity of MHCC-97H cells at different time points after knockdown of Igκ. **F** Representative plots and quantification of apoptotic MHCC-97H cells after knockdown of Igκ by flow cytometric analysis. **G - I** Wound healing assay (G) and migration transwell assay (H-I) were used to detect the migration ability of MHCC-97H cells with Igκ knockdown by siRNA or knockout by Igκ gRNA. Quantification of relative wound healing area and migrated cell counts are shown (low right). Scale bar, 100 μm (left), 50 μm (right). Data are presented as mean ± SD. (n = 3). * *p <* 0.05, ** *p <* 0.01, *** *p <* 0.001, **** *p <* 0.0001, ns, not significant.

**Figure S3**


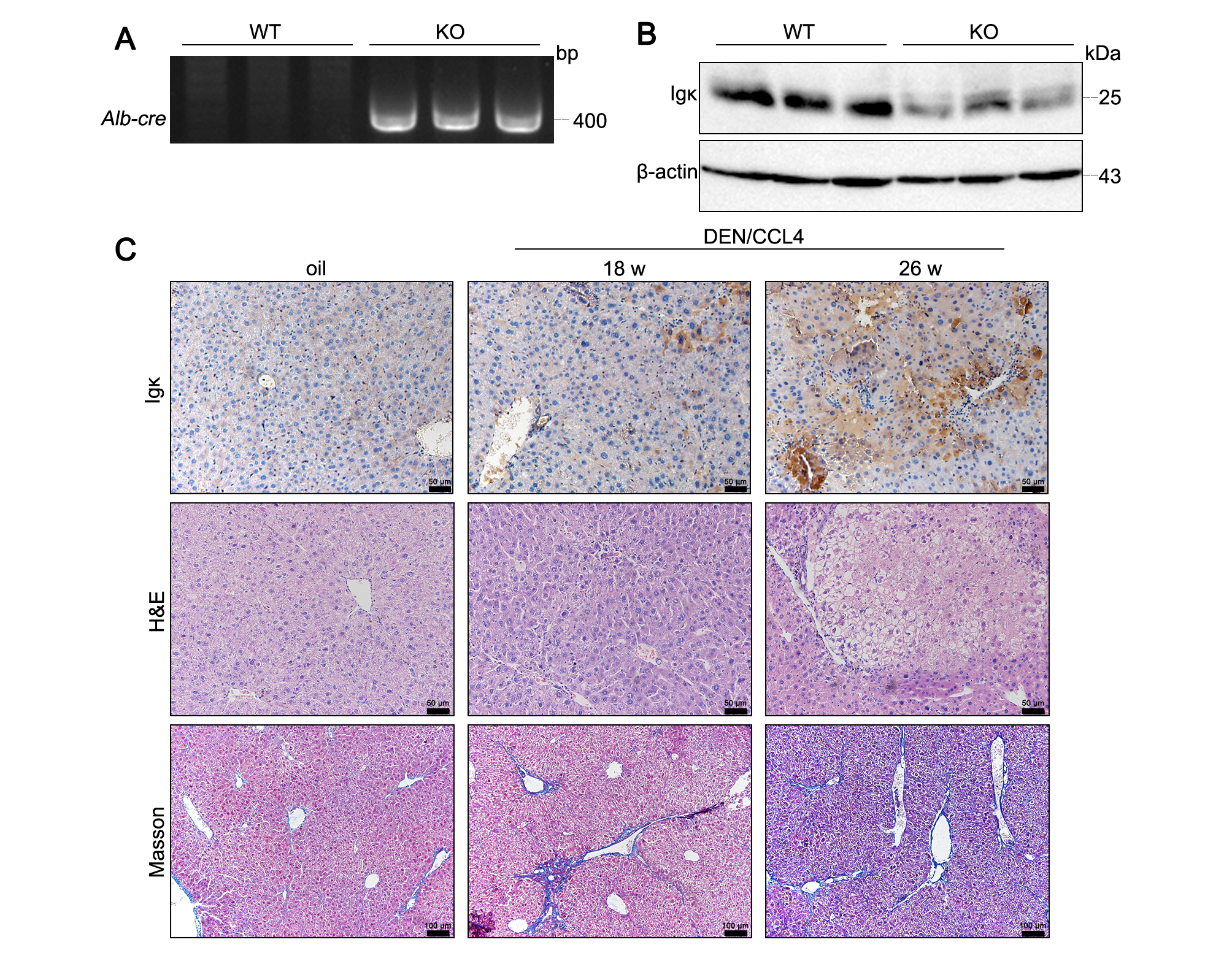


**Fig. S3** **The morphology and Igκ expression in mice liver tissues at different stages of HCC.** **A** PCR analysis of *Alb-cre* transcript in liver tissue of wild type (WT) and knockout (KO) mice (n = 3). **B** Western blot analysis of Igκ protein in liver tissue of WT and KO mice (n = 3). **C** Liver sections of WT mice were stained with H&E and Masson staining, as well as immunohistochemistry analysis of Igκ at the 18th week and 26th week. The group of oil as a negative control. Representative images are shown. Scale bar, 50 μm and 100 μm.

**Figure S4**


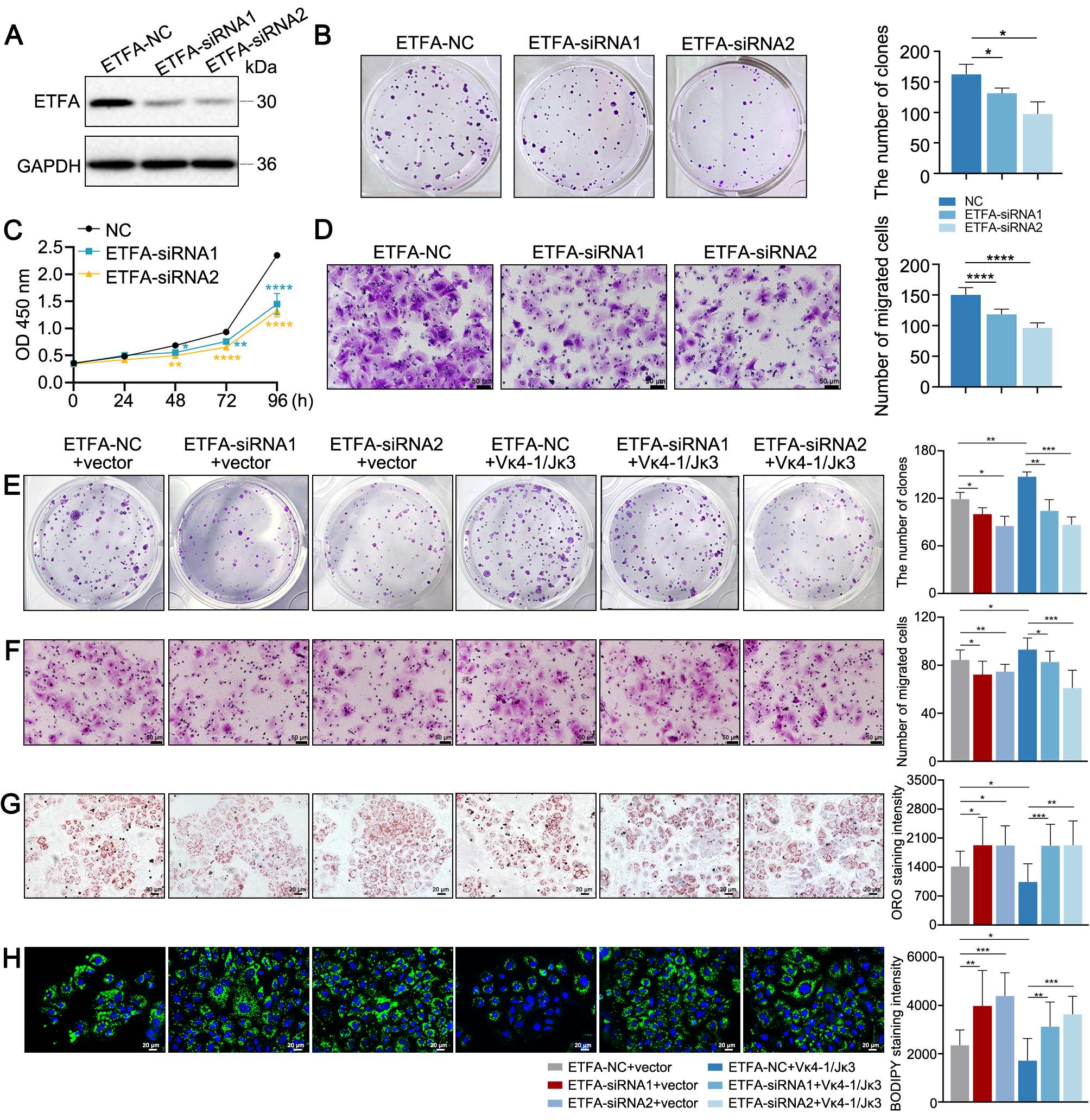


**Fig. S4 Loss of ETFA impairs mitochondrial respiration and fatty acid β-oxidation. A** The levels of ETFA protein in Huh7 cells treated with ETFA siRNA were detected by western blot analysis. **B** The colony formation assay was conducted to detect the proliferation capacity of Huh7 cells after ETFA knockdown. The quantification of the number of clones are shown right (n = 3). **C** CCK-8 assay were conducted to detect the proliferation capacity of Huh7 cells at different time points after knockdown of ETFA (n = 3). **D** Transwell assay was used to detect the migration ability of Huh7 cells with ETFA knockdown by siRNA. Quantification of clone numbers and migrated cell counts are shown right (n = 3). Scale bar, 50 μm. **E - F** Huh7 cells treated with siRNA against ETFA and transfected with pcDNA3.1-vector or pcDNA3.1-Vκ4-1/Jκ3-Igκ. These cells were then subjected to colony formation and transwell assays. The quantification of clone numbers and migrated cell counts are shown right (n = 3). Scale bar, 50 μm. **G** Representative images of Oil Red O staining in Huh7 cells after ETFA knockdown and treated with pcDNA3.1-vector or pcDNA3.1-Vκ4-1/Jκ3-Igκ are presented. The quantification of ORO staining intensity is shown right (n = 3). Scale bar, 20 μm. **H** Huh7 cells treated with siRNA against ETFA and then with pcDNA3.1-vector or pcDNA3.1-Vκ4-1/Jκ3-Igκ were subjected to BODIPY^TM^ 493/503 staining. The fluorescence intensity was analyzed using fluorescence microscopy. The quantification of the BODIPY^TM^ 493/503 staining intensity is shown right (n = 3). Scale bar, 10 μm. Data are presented as mean ± SD. * *p <* 0.05, ** *p <* 0.01, *** *p <* 0.001, **** *p <* 0.0001, ns, not significant.

**Table S1** The sequence of siRNA, shRNA and gRNA

| Gene | Sequence |
| --- | --- |
| Igκ siRNA1 | Sense：5’- AGGCCAAAGUACAGUGGAA-3’ |
| Igκ siRNA2 | Sense：5’- GACAGCACCUACAGCCUCATT-3’ |
| Igκ non-silencing siRNA | Sense：5’- GUAUGACAACAGCCUCAAGTT -3’ |
| ETFA-siRNA1 | Sense：5’- GAGAACUAUUUAUGCAGGAAA-3’ |
| ETFA-siRNA2 | Sense：5’- GCUUGACCAGAAAUUAACAAA-3’ |
| ETFA non-silencing siRNA | Sense：5’- UUCUCCGAACGUGUCACGUTT-3’ |
| Igκ gRNA1 | F primer 5’-CACCGGGTGGATAACGCCCTCCAAT-3’  R primer 5’-AAACATTGGAGGGCGTTATCCACCC-3’ |
| Igκ gRNA2 | F primer 5’-CACCGCCTGGGAGTTACCCGATTGG-3’  R primer 5’- AAACCCAATCGGGTAACTCCCAGGC-3’ |
| Igκ shRNA | CCGGAGGCCAAAGTACAGTGGAATTCAAGAGA  TTCCACTGTACTTTGGCCTTTTTT |
| Non-silencing shRNA | TTCTCCGAACGTGTCACGTTTCAAGAGA  ACGTGACACGTTCGGAGAATTTTTT |

**Table S2** The sequence of primers used for PCR

| Gene  name |  | Primer sequence 5’-3’ | Product size (bp) |
| --- | --- | --- | --- |
| *Igκ* | External Sense primer | GACATCGAGCTCACCCAGTCTCC |  |
|  | External antisense primer | CGGGAAGATGAAGACAGATGGTGC | 360-380 |
|  | Internal Sense primer | GAAATTGAGCTCACGCAGTCTCCA |  |
|  | Internal antisense primer | TGGTGCAGCCACAGTTCGTT | 340-360 |
| *PPARA* | Sense primer | TCGGCGAGGATAGTTCTGGAAG |  |
|  | Antisense primer | GACCACAGGATAAGTCACCGAG | 137 |
| *ACOX1* | Sense primer | GGCGCATACATGAAGGAGACCT |  |
|  | Antisense primer | AGGTGAAAGCCTTCAGTCCAGC | 112 |
| *ACSL1* | Sense primer | ATCAGGCTGCTCATGGATGACC |  |
|  | Antisense primer | AGTCCAAGAGCCATCGCTTCAG | 133 |
| *EHHADH* | Sense primer | CGGAGCATCGTGGAAAACAGCA |  |
|  | Antisense primer | CCGAGTCTACAGCAATCACAGG | 131 |
| *ACOT1* | Sense primer | GGGTTTTGCTGTGATGGCTCTG |  |
|  | Antisense primer | CAGCCCAACTCCTGGACCTTTT | 142 |
| *CPT2* | Sense primer | GCAGATGATGGTTGAGTGCTCC |  |
|  | Antisense primer | AGATGCCGCAGAGCAAACAAGTG | 99 |
| *PGC1A* | Sense primer | CCAAAGGATGCGCTCTCGTTCA |  |
|  | Antisense primer | CGGTGTCTGTAGTGGCTTGACT | 147 |
| *ACADM* | Sense primer | AGAACCTGGAGCAGGCTCTGAT |  |
|  | Antisense primer | GGATCTGGATCAGAACGTGCCA | 150 |
| *CPT1A* | Sense primer | GATCCTGGACAATACCTCGGAG |  |
|  | Antisense primer | CTCCACAGCATCAAGAGACTGC | 142 |
| *CPT1B* | Sense primer | TGTATCGCCGTAAACTGGACCG |  |
|  | Antisense primer | TGTCTGAGAGGTGCTGTAGCAC | 147 |
| *GAPDH* | Sense primer | CAAGGTCATCCATGACAACTTTG |  |
|  | Antisense primer | GTCCACCACCCTGTTGCTGTAG | 496 |

**Table S3** The list of antibodies

| Antigen | Product code | Supplier |
| --- | --- | --- |
| Igκ | 14678-1-AP | Proteintech |
| ETFA | 12262-1-AP | Proteintech |
| NDUFS3 | 15066-1-AP | Proteintech |
| SDHA | 14865-1-1AP | Proteintech |
| UQCRC2 | 14742-1-AP | Proteintech |
| COX1 | BA2149 | Biomed |
| IgG | SAB3701275 | SIGMA |
| His-Tag | 66005-1-Ig | Proteintech |
| Flag-Tag | 66008-2-Ig | Proteintech |
| Ki67 | 12202T | CST |
| GAPDH | TA-08 | ZSGB-Bio |
| Mouse IgG | SP031 | Solarbio |

**Table S4** The percentage of Vκ/Jκ rearrangement patterns in HCC cell lines and HCC patients

| Rearrangement pattern | Cell lines | | HCC Patients | | | | | |
| --- | --- | --- | --- | --- | --- | --- | --- | --- |
|  | Huh7 | MHCC-97H | Patient  1 | Patient  2 | Patient  3 | Patient  4 | Patient  5 | Patient  6 |
| Vκ4-1/Jκ3 | 83.4% | 71.4% | 62.5% | 75% | 70% | 77.8% | 62.5% | 42.8% |
| Vκ1-5/Jκ3 | 16.6% | 14.3% |  | 12.5% |  |  |  | 14.3% |
| Vκ1-12/Jκ1 |  |  |  |  |  |  |  | 14.3% |
| Vκ1-39/Jκ1 |  | 14.3% |  | 12.5% |  |  |  |  |
| Vκ2-28/Jκ1 |  |  | 12.5% |  |  |  |  |  |
| Vκ3-11/Jκ2 |  |  |  |  |  |  |  | 14.3% |
| Vκ3-11/Jκ4 |  |  | 12.5% |  |  |  |  |  |
| Vκ3-15/Jκ1 |  |  |  |  |  | 11.1% |  | 14.3% |
| Vκ3-15/Jκ2 |  |  |  |  |  |  | 25% |  |
| Vκ3-15/Jκ4 |  |  | 12.5% |  | 10% |  |  |  |
| Vκ3-20/Jκ1 |  |  |  |  | 20% | 11.1% | 12.5% |  |

**Table S5** *IGK* expression in human primary HCC cells or HCC cell lines from GEO Datasets

| Dataset | Platform | Title | PMID | Source | *IGK* |
| --- | --- | --- | --- | --- | --- |
| GSE10393 | GPL3921 | Integrative Transcriptome Analysis Reveals Common Molecular Subtypes of Human Hepatocellular Carcinoma  (HT-HG_U133A) | 19723656 | Patients | *IGKC*  *IGKV1-5 IGKV1D-13 IGKV2-24* |
| GSE41804 | GPL570 | Hepatic gene expression of HCV related Hepatocellular carcinoma and non-cancerous tissue with Il28B rs8099917 TT genotype and TG/GG genotype | 23426277 | Patients | *IGKC*  *IGKV1-17 IGKV1-5*  *IGKV2-28*  *IGKV4-1 IGKV1-37*  *IGKV1OR2-108*  *IGKV1OR2-2*  *IGKV1OR10-1* |
| GSE4731 | GPL96 | Effect of GPI-PLD on global gene expression in HepG2 cells | 16595594 | HepG2 |  |
| GSE23031 | GPL570 | Ribavirin Treated Huh7.5.1 Cells | 21254160 | Huh7 |  |
| GSE29084 | GPL570 | Expression Data from HNF4a RNAi Knockdown in HepG2 cells | 20054869 | HepG2 |  |

**Table S6** A list of potential proteins that are interacted with Vκ4-1/Jκ3-Igκ

| Gene name | Protein name | Number of proteins | Peptides | Score | PSMs |
| --- | --- | --- | --- | --- | --- |
| ETFA | Electron transfer flavoprotein subunit α | 6 | 2 | 44.577 | 12 |
| DAZAP1 | DAZ-associated protein 1 | 4 | 2 | 4.4437 | 5 |
| ACAT1 | Acetyl-CoA acetyltransferase | 2 | 3 | 4.3913 | 3 |
| PDHB | Pyruvate dehydrogenase E1 component subunit beta | 3 | 2 | 4.1369 | 2 |
| RTCB | tRNA-splicing liganse RtcB homolog | 1 | 2 | 3.7627 | 2 |
| ZMPSTE24 | CAAX prenyl protease 1 homolog | 1 | 2 | 3.1904 | 4 |
| PIP | Prolactin-inducible protein | 1 | 2 | 3.1014 | 5 |
| CTSZ | Cathepsin Z | 1 | 2 | 2.7507 | 2 |
| SRPRB | Signal recognition particle receptor subunit beta | 2 | 2 | 2.6685 | 4 |
| TIA1 | Nucleolysin TIA-1 isoform p40 | 5 | 4 | 2.6222 | 4 |
| SLC3A2 | 4F2 cell-surface antigen heavy chain | 7 | 3 | 2.5465 | 6 |
| RPS4X | 40S ribosomal protein, X isoform | 4 | 4 | 1.6251 | 4 |
| ATP5H | ATP synthase subunit D | 2 | 2 | 1.5132 | 5 |
